# Targeted degradation of oncogenic KRAS^G12V^ triggers antitumor immunity in lung cancer models

**DOI:** 10.1172/JCI174249

**Published:** 2024-12-24

**Authors:** Dezhi Li, Ke Geng, Yuan Hao, Jiajia Gu, Saurav Kumar, Annabel T. Olson, Christina C. Kuismi, Hye Mi Kim, Yuanwang Pan, Fiona Sherman, Asia M. Williams, Yiting Li, Fei Li, Ting Chen, Cassandra Thakurdin, Michela Ranieri, Mary Meynardie, Daniel S. Levin, Janaye Stephens, Alison Chafitz, Joy Chen, Mia S. Donald-Paladino, Jaylen M. Powell, Ze-Yan Zhang, Wei Chen, Magdalena Ploszaj, Han Han, Shengqing Stan Gu, Tinghu Zhang, Baoli Hu, Benjamin A. Nacev, Medard Ernest Kaiza, Alice H. Berger, Xuerui Wang, Jing Li, Xuejiao Sun, Yang Liu, Xiaoyang Zhang, Tullia C. Bruno, Nathanael S. Gray, Behnam Nabet, Kwok-Kin Wong, Hua Zhang

**Affiliations:** 1 Division of Hematology and Medical Oncology, Laura and Isaac Perlmutter Cancer Center, New York University Langone Health, New York, New York, USA.; 2 Applied Bioinformatics Laboratories, Office of Science and Research, New York University Grossman School of Medicine, New York, New York, USA.; 3 Hillman Cancer Center, University of Pittsburgh Medical Center (UPMC), Pittsburgh, Pennsylvania, USA.; 4 Human Biology Division, Fred Hutchinson Cancer Center, Seattle, Washington, USA.; 5 Department of Immunology, University of Pittsburgh, Pittsburgh, Pennsylvania, USA.; 6 School of Medicine, Tsinghua University, Beijing, China.; 7 Department of Pathology, School of Basic Medical Sciences, and; 8 Frontier Innovation Center, School of Basic Medical Sciences, Fudan University, Shanghai, China.; 9 Department of Radiation Oncology, New York University Grossman School of Medicine, New York, New York, USA.; 10 Division of Pulmonary Medicine, Department of Pediatrics, UPMC Children’s Hospital of Pittsburgh and University of Pittsburgh, Pittsburgh, Pennsylvania, USA.; 11 Department of Hematopoietic Biology and Malignancy, University of Texas MD Anderson Cancer Center, Houston, Texas, USA.; 12 Department of Chemical and Systems Biology, Chem-H and Stanford Cancer Institute, Stanford School of Medicine, Stanford University, Stanford, California, USA.; 13 Department of Neurological Surgery,; 14 Department of Medicine, Division of Hematology/Oncology, and; 15 Department of Pathology, University of Pittsburgh School of Medicine, Pittsburgh, Pennsylvania, USA.; 16 Department of Bioengineering, University of Illinois Urbana-Champaign, Urbana, Illinois, USA.; 17 Department of Oncological Sciences, Huntsman Cancer Institute, University of Utah, Salt Lake City, Utah, USA.; 18 Department of Pharmacology, University of Washington, Seattle, Washington, USA.

**Keywords:** Immunology, Oncology, Lung cancer

## Abstract

Kirsten rat sarcoma viral oncogene homolog (*KRAS*) is the most frequently mutated oncogene in lung adenocarcinoma, with *G12C* and *G12V* being the most predominant forms. Recent breakthroughs in KRAS^G12C^ inhibitors have transformed the clinical management of patients with the *G12C* mutation and advanced our understanding of the function of this mutation. However, little is known about the targeted disruption of KRAS^G12V^, partly due to a lack of specific inhibitors. Here, we leverage the degradation tag (dTAG) system to develop a *KRAS^G12V^-*transgenic mouse model. We explored the therapeutic potential of KRAS^G12V^ degradation and characterized its effect on the tumor microenvironment (TME). Our study reveals that degradation of KRAS^G12V^ abolished lung and pancreatic tumors in mice and caused a robust inhibition of KRAS-regulated cancer-intrinsic signaling. Importantly, targeted degradation of KRAS^G12V^ reprogrammed the TME toward a stimulatory milieu and drove antitumor immunity, elicited mainly by effector and cytotoxic CD8^+^ T cells. Our work provides insights into the effect of KRAS^G12V^ degradation on both tumor progression and the immune response, highlighting degraders as a powerful strategy for targeting *KRAS*-mutant cancers.

## Abstract 2

Targeted degradation of KRAS G12V reprograms the tumor microenvironment and triggers antitumor immunity.

## Graphical abstract

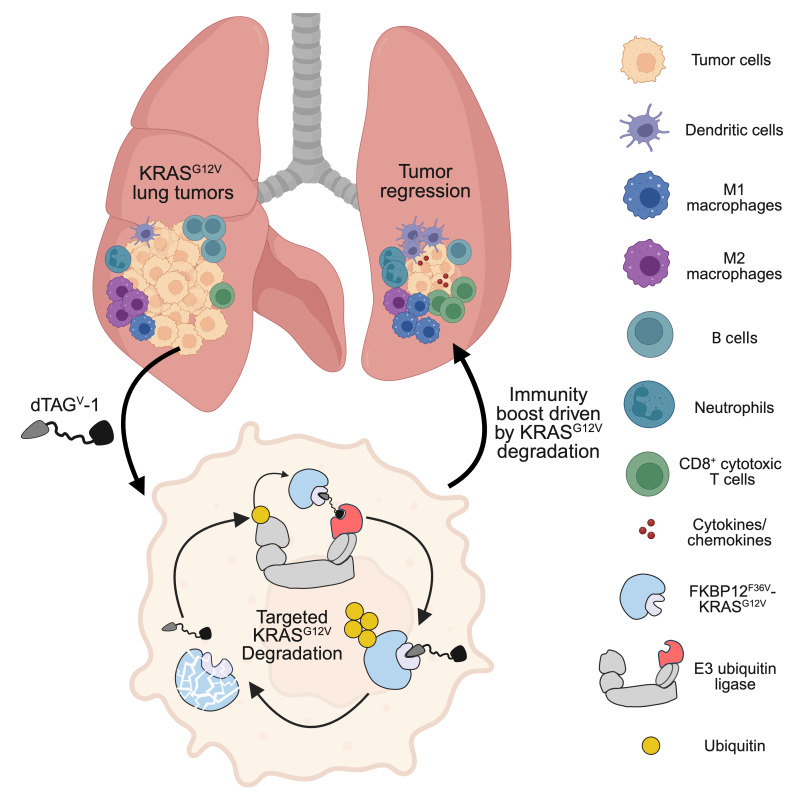

## Introduction

Non–small cell lung cancer (NSCLC) is one of the leading causes of cancer deaths worldwide (1). Kirsten rat sarcoma viral oncogene homolog (*KRAS*) is the most frequently mutated oncogene in lung adenocarcinoma, the most common subtype of NSCLC (2). Approximately 30% of patients with lung adenocarcinoma harbor *KRAS* mutations, which are most commonly *G12C* and *G12V* (3). Direct targeting of KRAS has been historically difficult until the recent development of KRAS^G12C^*-*specific inhibitors including ARS-1620, AMG-510, and MRTX849 (4–7). These inhibitors have shown strong antitumor effects in *KRAS^G12C^*-mutated lung adenocarcinoma preclinical models and in patients (6, 8, 9). Notably, on the basis of the positive clinical benefit observed in large clinical trials, the FDA recently approved AMG-510 (sotorasib) for the treatment of patients with KRAS^G12C^-mutated NSCLC. Despite this remarkable breakthrough, sotorasib demonstrates an approximately 30% response rate in patients with lung cancer (9, 10), with a rapid emergence of drug resistance (11–13). Furthermore, in stark contrast to the substantial advances in KRAS^G12C^ drug discovery, there are currently no approved specific inhibitors for KRAS^G12V^. As drug discovery efforts focus on KRAS^G12V^, an improved understanding of the biological consequences of KRAS^G12V^ disruption for tumor-intrinsic signaling and the tumor microenvironment (TME) in vivo is necessary.

Targeted protein degradation has emerged as a powerful therapeutic approach to target oncogenic drivers (14–17). Proteolysis-targeting chimeras (PROTACs) are a class of small-molecule degraders that bind a target protein and E3 ligase, leading to target protein ubiquitination and rapid proteasome-mediated degradation (18). PROTACs are advantageous over inhibitors because of their ability to abolish all protein activity including scaffolding functions (19, 20). We and others have endeavored to develop PROTACs to degrade KRAS^G12C^, which has proven to be challenging (21, 22). While PROTACs such as LC-2 are capable of degrading KRAS^G12C^, the benefits and liabilities of KRAS degradation in vivo remain unclear (22). Furthermore, although pan-KRAS degraders are in preclinical development (23–25) and KRAS^G12D^ degraders are in clinical trials (NCT05382559) (26), the consequences of targeted KRAS^G12V^ degradation in immune-competent models and the characterization of KRAS^G12V^-selective degraders remain largely unexplored. Prior to investment in the development of degraders, strategies to model the pharmacological degradation of drug targets are necessary.

As a solution to this challenge, we developed a versatile approach known as the degradation tag (dTAG) system to deplete tagged proteins in vitro and in vivo (27, 28). In this approach, a protein is expressed with an FKBP12^F36V^ tag and is targeted for degradation using dTAG molecules that recruit an E3 ubiquitin ligase. We previously demonstrated that the dTAG system can be effectively used to study the consequences of rapid and selective KRAS^G12V^ degradation in several cellular models (27–29). We and others have extensively applied the dTAG system to degrade diverse targets including oncoproteins, transcription factors, chromatin regulators, and kinases, illustrating the utility of the dTAG system for drug target validation and discovery (27, 28, 30, 31).

Mouse models are invaluable for understanding the biology of lung cancer, identifying potential therapeutic targets, and testing new treatments in a preclinical setting. Previous studies utilizing *KRAS^G12V^* mouse models have advanced our understanding of KRAS^G12V^-driven lung cancer and nominated new potential therapeutic approaches (32–35). In this study, to develop a platform for drug target validation in vivo, we advanced the dTAG system to establish a genetically engineered mouse model (GEMM) harboring *KRAS^G12V^* that can be degraded with high specificity and speed. This powerful model enabled us to comprehensively characterize the therapeutic potential of degrading KRAS^G12V^. Utilizing this *KRAS^G12V^* GEMM, we were able to dissect the tumor-intrinsic responses as well as extrinsic effects, including the effect on the TME upon degradation of KRAS^G12V^. Our findings offer strong evidence for the promise of developing degraders targeting mutant KRAS in cancer and also establish an in vivo platform for drug target discovery and validation.

## Results

### Establishing a GEMM for targeted degradation of KRAS^G12V^ in lung cancer.

**Figure 1:**
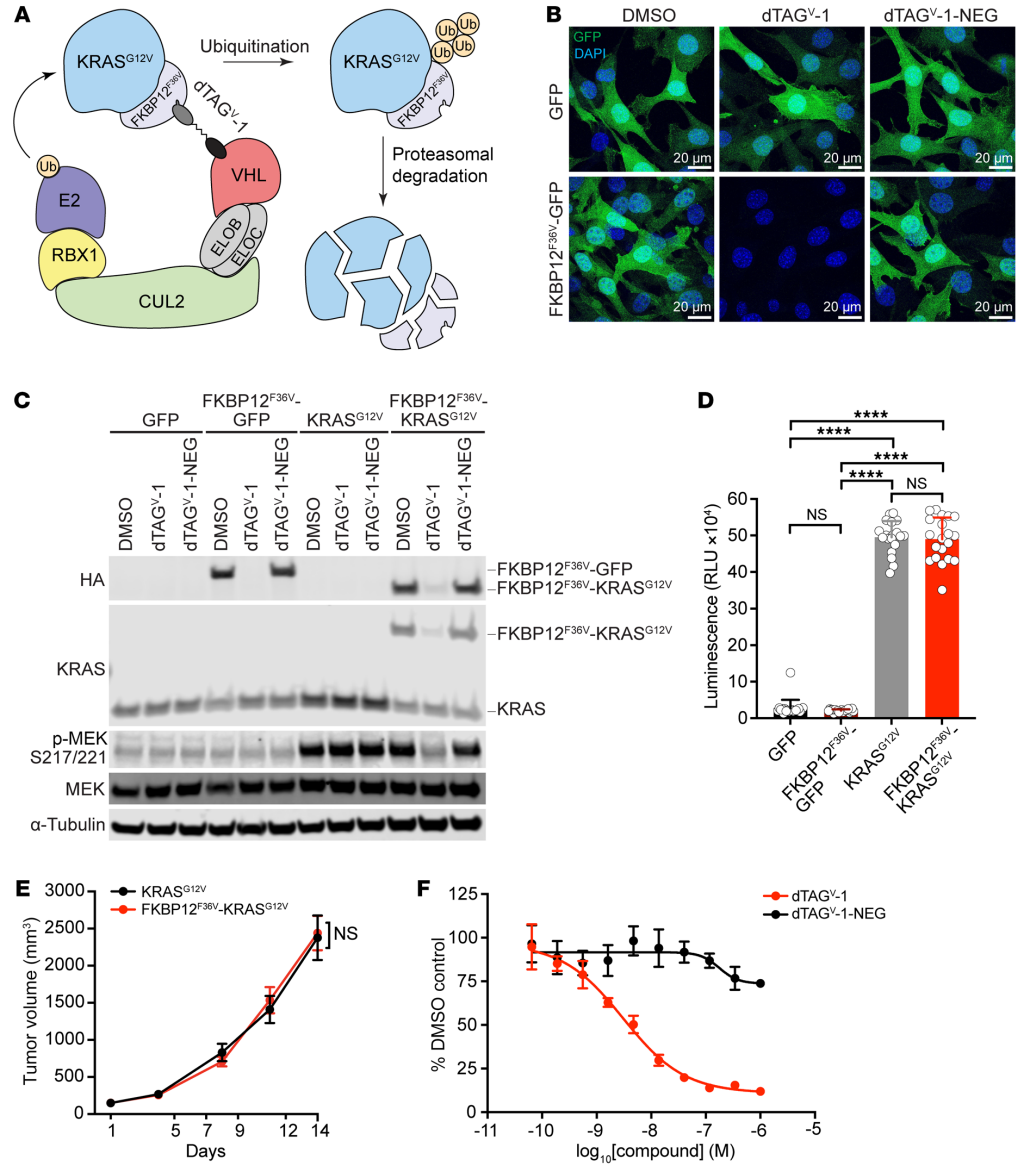
Validation of targeted degradation of KRAS^G12V^ using the dTAG system. (**A**) Schematic of the dTAG system showing that dTAG^V^-1 recruits the VHL E3 ubiquitin ligase to induce targeted degradation of FKBP12^F36V^-KRAS^G12V^. The schematic was created using BioRender.com. CUL2, Cullin 2; ELOB, Elongin B; ELOC, Elongin C; E2, ubiquitin-conjugating enzyme; RBX1, RING-box protein 1; Ub, ubiquitin. (**B**) Representative images of NIH/3T3 cells expressing GFP or FKBP12^F36V^-GFP that were treated with DMSO, 500 nM dTAG^V^-1, or 500 nM dTAG^V^-1-NEG for 8 hours. Scale bars: 20 μm. Data are representative of 3 independent experiments. (**C**) Immunoblot analysis of HA to detect FKBP12^F36V^-GFP or FKBP12^F36V^-KRAS^G12V^, KRAS, p-MEK, MEK, and α-tubulin in NIH/3T3 cells expressing GFP, FKBP12^F36V^-GFP, KRAS^G12V^, or FKBP12^F36V^-KRAS^G12V^ that were treated with DMSO, 500 nM dTAG^V^-1, or 500 nM dTAG^V^-1-NEG for 8 hours. Data are representative of 3 independent experiments. (**D**) Antiproliferation of NIH/3T3 cells expressing GFP, FKBP12^F36V^-GFP, KRAS^G12V^, or FKBP12^F36V^-KRAS^G12V^ cultured as ultra-low adherent 3D spheroid suspensions for 144 hours. Data are presented as the mean ± SD of 20 biologically independent samples and are representative of 3 independent experiments. (**E**) Volume changes of tumors from NIH/3T3 expressing KRAS^G12V^ or FKBP12^F36V^-KRAS^G12V^ that were s.c. injected into mice. Data are presented as the mean ± SEM of 10 per group. (**F**) DMSO-normalized proliferation of NIH/3T3 cells expressing FKBP12^F36V^-KRAS^G12V^ cultured as ultra-low adherent 3D spheroid suspensions and treated with the indicated compounds for 120 hours. Data are presented as the mean ± SD of 4 biologically independent samples and are representative of 3 independent experiments. ****P < 0.0001 (**D**) and NS (**D** and **E**), by 1-way ANOVA with post hoc Tukey’s test (**D**) or 2-tailed Student’s *t* test (**E**).

Chemical genetic degron strategies for achieving rapid, selective, and robust target protein loss have emerged as powerful approaches for biological study and drug target validation (31, 36). However, there are limited generalizable targeted degradation strategies available to study drug target loss in vivo. KRAS^G12V^ is an ideal drug target to evaluate the consequences of targeted degradation. Critically, the effect of KRAS^G12V^ protein degradation on tumorigenesis, intrinsic signaling, and the TME is poorly understood, which is due to limited relevant mouse models and specific KRAS^G12V^ inhibitors or degraders. To address these challenges, we set out to leverage the dTAG system (27, 28) to establish a GEMM harboring *KRAS^G12V^* that can be rapidly and selectively degraded (detailed in Methods). In our approach, dTAG molecules bind an FKBP12^F36V^ tag and recruit an E3 ubiquitin ligase in proximity to induce FKBP12^F36V^–fusion protein degradation (Figure 1A and Supplemental Figure 1A; supplemental material available online with this article; https://doi.org/10.1172/JCI174249DS1). We previously demonstrated that our dTAG molecules known as dTAG^V^-1 and dTAG-13, which recruit von Hippel-Lindau (VHL) or cereblon (CRBN), respectively, are selective and degrade KRAS^G12V^ in several cellular models, including pancreatic ductal adenocarcinoma cell lines (27, 28). We also demonstrated that these dTAG molecules display suitable pharmacokinetic (PK) and pharmacodynamic (PD) properties to degrade tagged fusions in xenograft mouse models (27, 28, 37). Recent work has further confirmed the tolerability of dTAG molecules in vivo and has shown that dTAG molecules effectively degrade FKBP12^F36V^-tagged proteins in embryonic stages of mouse development (38), in several mouse organs (39), and in patient-derived xenograft models (40).

Building on our prior work, we aimed to confirm that the FKBP12^F36V^-KRAS^G12V^ protein is functional and that it elicits comparable oncogenic responses to untagged KRAS^G12V^ in vitro and in vivo. We first utilized NIH/3T3 cells, a commonly used model for testing oncogenic driver genes, and ectopic expression of GFP or FKBP12^F36V^-GFP as controls (Figure 1B), as well as KRAS^G12V^ and FKBP12^F36V^-KRAS^G12V^ (Figure 1C). The FKBP12^F36V^-GFP and FKBP12^F36V^-KRAS^G12V^ fusions also included hemagglutinin (HA) tags to facilitate monitoring of GFP and KRAS^G12V^ levels. Importantly, comparable hyperactivation of phosphorylated MEK (p-MEK), a key component of oncogenic KRAS^G12V^ downstream signaling, was observed upon the expression of KRAS^G12V^ and FKBP12^F36V^-KRAS^G12V^ (Figure 1C). We next confirmed the effectiveness of the recruitment of VHL to degrade FKBP12^F36V^-GFP or FKBP12^F36V^-KRAS^G12V^ and reverse these responses. We observed that dTAG^V^-1 treatment resulted in the robust degradation of FKBP12^F36V^-GFP (Figure 1, B and C) and FKBP12^F36V^-KRAS^G12V^ (Figure 1C), with no effect on untagged GFP or KRAS^G12V^ levels, highlighting the specificity of dTAG^V^-1 toward FKBP12^F36V^-tagged fusions. The degradation of FKBP12^F36V^-KRAS^G12V^ rapidly reversed this aberrantly activated p-MEK response back to baseline levels (Figure 1C). Furthermore, dTAG^V^-1-NEG, a control dTAG molecule that can bind to FKBP12^F36V^ but not recruit VHL, did not degrade FKBP12^F36V^-GFP or FKBP12^F36V^-KRAS^G12V^ or alter p-MEK levels (Figure 1, B and C).

Next, we evaluated the oncogenic potential of KRAS^G12V^ or FKBP12^F36V^-KRAS^G12V^ in vitro and in vivo. While NIH/3T3 cells expressing GFP or FKBP12^F36V^-GFP were unable to proliferate as 3D spheroids, expression of KRAS^G12V^ or FKBP12^F36V^-KRAS^G12V^ led to 3D spheroid formation and a significant growth advantage compared with their counterparts in vitro (Figure 1D). There was no difference in the kinetics of 3D spheroid formation between KRAS^G12V^ and FKBP12^F36V^-KRAS^G12V^ in vitro (Figure 1D). To further examine their tumorigenesis in vivo, NIH/3T3 cells expressing KRAS^G12V^ or FKBP12^F36V^-KRAS^G12V^ were injected s.c. into the flanks of mice, and tumor volumes were measured. Consistently, the kinetics of tumorigenesis were comparable between KRAS^G12V^ and FKBP12^F36V^-KRAS^G12V^ in vivo, supporting the notion that the FKBP12^F36V^ tag did not alter KRAS^G12V^ function (Figure 1E). Importantly, dTAG^V^-1 treatment robustly diminished the proliferation and viability of NIH/3T3 cells expressing FKBP12^F36V^-KRAS^G12V^ (Figure 1F). With our goal to evaluate targeted KRAS^G12V^ degradation in lung cancer models, we next confirmed these observations in human lung epithelial cells (AALE cells) (Supplemental Figure 1B). We have previously shown that KRAS^G12V^ transforms AALE cells and increases p-MEK levels (41, 42). Similar to the results with NIH/3T3 cells, compared with FKBP12^F36V^-GFP, we observed that ectopic expression of FKBP12^F36V^-KRAS^G12V^ in AALE cells led to elevated p-MEK levels and the formation of 3D spheroids (Supplemental Figure 1, B and C). We observed pronounced degradation of FKBP12^F36V^-KRAS^G12V^ upon treatment with dTAG^V^-1, leading to a reversal of p-MEK back to baseline levels and diminished proliferation as 3D spheroids (Supplemental Figure 1, B and D). Together, these results show that the FKBP12^F36V^ tag did not affect the functionality of the oncoprotein or alter the kinetics of KRAS^G12V^-induced tumorigenesis and validate the effectiveness of targeted degradation of FKBP12^F36V^-KRAS^G12V^ by dTAG^V^-1.

**Figure 2:**
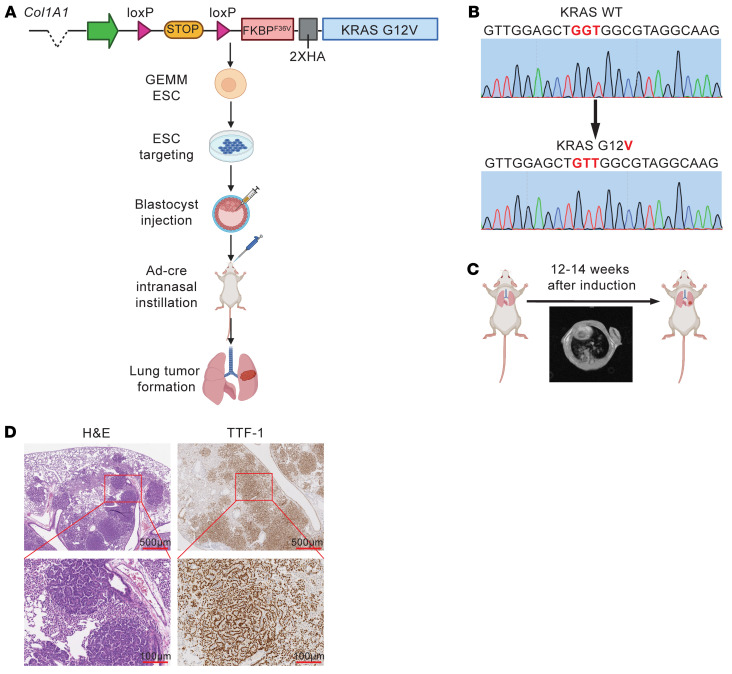
Establishing a GEMM for targeted degradation of KRAS^G12V^ in lung cancer. (**A**) Schematic showing the design of the *FKBP12^F36V^*-*KRAS^G12V^* GEMM. The schematic was created using BioRender.com. (**B**) Genomic sequencing confirmation of the *KRAS^G12V^* mutation in the GEMM. (**C**) MRI was performed to detect lung tumor nodules 12–14 weeks after adenovirus-carrying Cre-recombinase delivery. (**D**) Representative images of H&E and IHC staining for TTF-1 in lung tumors from the *FKBP12^F36V^*-*KRAS^G12V^* GEMM. Scale bars: 500 μm (top panels) and 100 μm (bottom panels).

These results motivated our development of a transgenic lung cancer mouse model that enabled specific degradation of FKBP12^F36V^-KRAS^G12V^ using dTAG^V^-1. We first designed a targeting vector that included a PGK promoter and Lox-Stop-Lox cassette to allow for temporal and spatial control of gene expression, as we previously described (43) (Figure 2A). The transgene expression is controlled by the Lox-Stop-Lox cassette, which can be removed by Cre recombinase. *FKBP12^F36V^-KRAS^G12V^* cDNA was subcloned into the targeting vector (Figure 2A). We also included HA tags to enable monitoring of KRAS^G12V^ levels. After the targeting vector was electroporated into mouse embryonic stem cells (ESCs), these cells were engineered to allow single-copy transgene insertion at the *Col1A1* locus. Mouse ESC clones that carry the *FKBP12^F36V^-KRAS^G12V^* transgene were selected, expanded, and injected into C57BL/6 (B6) blastocysts, which gave rise to chimeras (Figure 2A). The chimeras were bred with WT B6 mice, and transgene-positive mice were genotyped and sequenced, and then bred for experiments (Figure 2B).

We next sought to examine whether a single allele of *FKBP12^F36V^-KRAS^G12V^* would give rise to lung adenocarcinoma modeling human disease in this model. Intranasal adenovirus-carrying Cre-recombinase was used to induce tumors in *FKBP12^F36V^-KRAS^G12V^* mice that were 6–8 weeks of age. Starting from 12–14 weeks after the induction, *FKBP12^F36V^-KRAS^G12V^* mice had visible lung tumors detected by MRI (Figure 2C). We then harvested mouse lungs from these *FKBP12^F36V^-KRAS^G12V^* tumor–bearing mice to perform histologic analysis. H&E staining revealed the morphology of tumors formed by *FKBP12^F36V^-KRAS^G12V^* cells resembled differentiated adenocarcinomas showing nuclear pleomorphisms including enlarged nuclei with prominent nucleoli (Figure 2D) (44). IHC staining for the lung adenocarcinoma–specific marker TTF-1 demonstrated strong nuclear expression, further confirming the presence of a primary pulmonary adenocarcinoma (Figure 2D). Our *FKBP12^F36V^-KRAS^G12V^* mouse strain developed lung adenocarcinomas with complete penetrance and a consistent latency period, comparable to previously reported *KRAS^G12V^* models (32–35). In summary, we successfully established a GEMM of *FKBP12^F36V^-KRAS^G12V^* lung adenocarcinoma development that can be utilized for targeted degradation with the dTAG system.

### dTAG^V^-1 effectively degrades KRAS^G12V^ and abolishes tumor growth in a KRAS^G12V^ GEMM.

**Figure 3:**
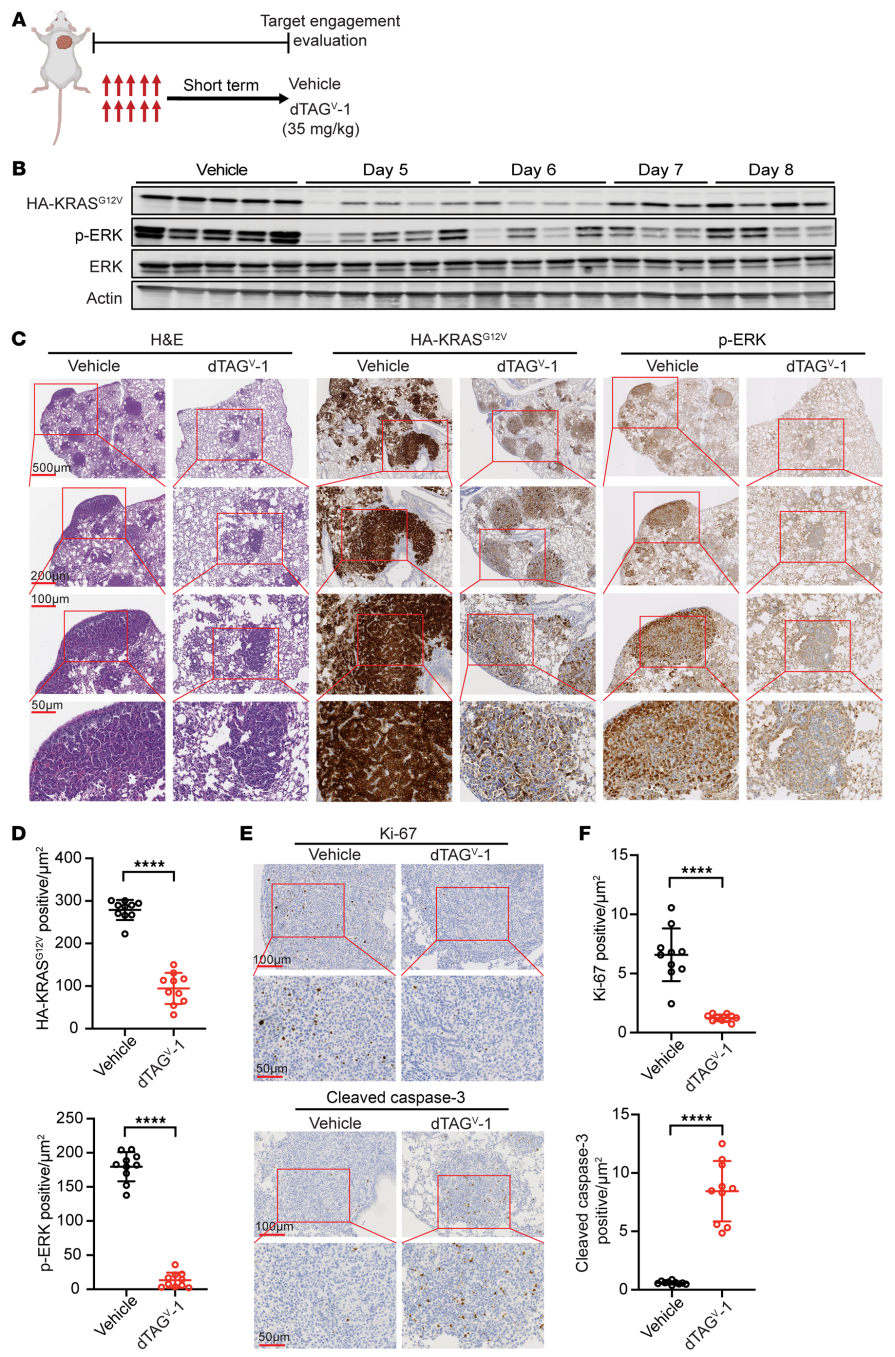
dTAG^V^-1 effectively degrades KRAS^G12V^ and inhibits downstream signaling in a *KRAS^G12V^*-driven lung cancer GEMM. (**A**) Schematic showing the in vivo dosing schedule for evaluation of target engagement and degradation. Mice were treated once daily with either vehicle or dTAG^V^-1 (35 mg/kg) for 5 days. The schematic was created using BioRender.com. (**B**) Immunoblot analysis of HA to detect FKBP12^F36V^-KRAS^G12V^, p-ERK, ERK, and actin in lung tumor nodules after the indicated treatment and duration (*n* = 3–5 per group). (**C**) Representative images of H&E and IHC staining for HA to detect FKBP12^F36V^-KRAS^G12V^ and p-ERK of lung tumors after the indicated treatment (*n* = 3 mice per group). Scale bars: 500 μm, 200 μm, 100 μm, and 50 μm (from top to bottom). (**D**) Quantification of HA to detect FKBP12^F36V^-KRAS^G12V^ and p-ERK^+^ staining after the indicated treatment. Data are presented as the mean ± SD of 10 representative areas from 3 mice per group. (**E**) Representative images of IHC staining for Ki-67 and cleaved caspase-3 in lung tumors after the indicated treatment. Scale bars: 100 μm (top panels) and 50 μm (bottom panels). (**F**) Quantification of Ki-67 and cleaved caspase-3^+^ staining after the indicated treatment. Data are presented as the mean ± SD of 10 representative areas from 3 mice per group. ****P < 0.0001, by 2-tailed Student’s *t* test (**D** and **F**).

We next focused on evaluating the acute and prolonged responses to dTAG-mediated degradation of FKBP12^F36V^-KRAS^G12V^. On the basis of our prior PK and PD studies (28), we treated a cohort of *FKBP12^F36V^-KRAS^G12V^* tumor–bearing mice with 35 mg/kg dTAG^V^-1 continuously for 5 days (formulation is described in Supplemental Methods and was performed as previously described in ref. 28), harvested the tumor nodules, and evaluated FKBP12^F36V^-KRAS^G12V^ degradation by monitoring the HA tag and downstream signaling (Figure 3A). Notably, we observed robust degradation of FKBP12^F36V^-KRAS^G12V^, with a concomitant decrease in downstream p-ERK signaling shown by Western blotting and IHC staining (Figure 3, B–D). To examine the duration of FKBP12^F36V^-KRAS^G12V^ degradation in vivo, we treated a separate cohort of tumor-bearing mice with dTAG^V^-1 continuously for 5 days. We then stopped compound administration and harvested tumors on days 5 (2 hours after the last dose), 6, 7, and 8. We observed that effective FKBP12^F36V^-KRAS^G12V^ degradation lasted for 72 hours following the last administration, before returning to levels comparable to those in the vehicle-treated group (Figure 3B and Supplemental Figure 2A). These results demonstrated successful target engagement and durable degradation of FKBP12^F36V^-KRAS^G12V^ by dTAG^V^-1. Furthermore, we examined the antiproliferative and apoptotic effects upon abrupt FKBP12^F36V^-KRAS^G12V^ loss after 5 days of dTAG^V^-1 treatment. IHC staining for the proliferation marker Ki-67 and the apoptosis marker cleaved caspase-3 showed that dTAG^V^-1 led to a significant decrease in Ki-67 levels and an increase in cleaved caspase-3 levels (Figure 3, E and F, and Supplemental Figure 2B). We next investigated the effects of acute FKBP12^F36V^-KRAS^G12V^ degradation on extracellular matrix component collagen using Masson’s trichrome staining. Interestingly, dTAG^V^-1 treatment caused a reduction in collagen matrices in tumor-bearing lungs (Supplemental Figure 2, C and D), suggesting a potential effect on the TME upon FKBP12^F36V^-KRAS^G12V^ degradation.

**Figure 4:**
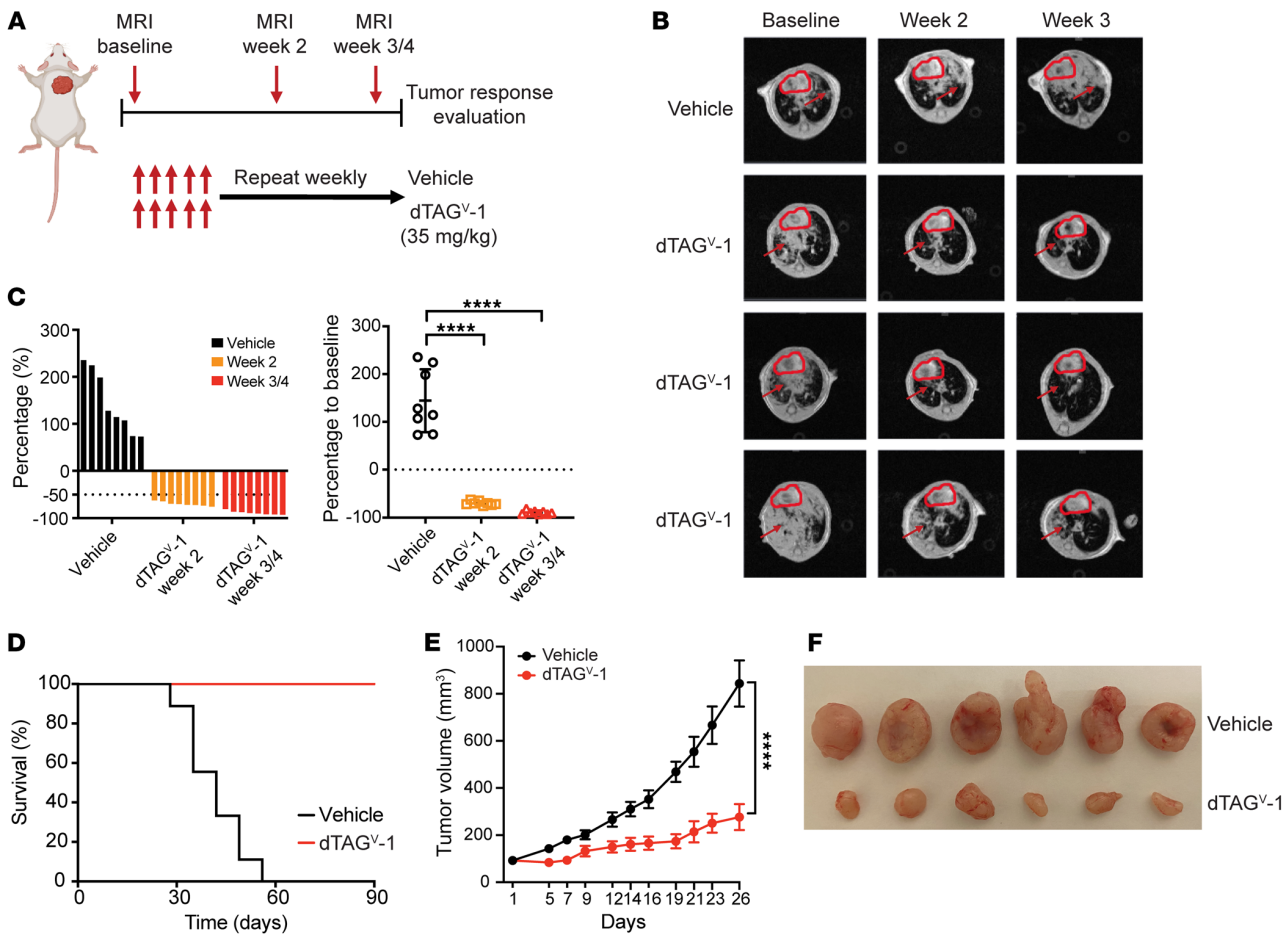
KRAS^G12V^ degradation abolishes tumor growth h in *KRAS^G12V^*-driven murine lung and pancreatic cancer models. (**A**) Schematic showing the in vivo dosing schedule for evaluation of long-term dTAG^V^-1 treatment. The schematic was created using BioRender.com. (**B**) Representative MRI scans (*n* = 1 vehicle-treated mouse and 3 dTAG^V^-1–treated mice) of tumor at baseline and 2 weeks and 3 weeks after treatment initiation. Red arrowheads indicate lung tumors; red circles indicate the heart. (**C**) Waterfall plot (left) and dot plot (right) showing changes in tumor volume compared with baseline after 2 or 3–4 weeks of treatment. Data are presented as the mean ± SD of 8 per group. (**D**) Kaplan-Meier survival curve of *FKBP12^F36V^*-*KRAS^G12V^* lung cancer mice after long-term treatment with vehicle or dTAG^V^-1 (*n* = 9 per group). (**E**) Volume changes of tumors from PATU-8902 FKBP12^F36V^-KRAS^G12V^; *KRAS^–/–^* cells that were s.c. injected into mice and treated with vehicle or dTAG^V^-1. Data are presented as the mean ± SEM of 12 per group. (**F**) Representative pancreatic tumors after the indicated treatment. ****P < 0.0001, by 1-way ANOVA with post hoc Dunnett’s test (**C**) or 2-tailed Student’s *t* test (**E**).

After confirming that dTAG^V^-1 successfully degraded FKBP12^F36V^-KRAS^G12V^ and inhibited oncogenic KRAS signaling, we proceeded to assess its effect on tumor growth in vivo. For this, a separate cohort of GEMM mice was induced by intranasal adenovirus-carrying Cre recombinase, and their tumor volumes were monitored and quantified using MRI. Once tumor volumes reached approximately 100 mm^3^, mice were randomized to vehicle or dTAG^V^-1 treatment groups (Figure 4A). All mice in the vehicle treatment group displayed aggressive disease progression after a 3-week period (Figure 4, B and C). In contrast, mice treated with dTAG^V^-1 showed a significant tumor response (Figure 4, B and C), with MRI imaging revealing a reduction in tumor burden of over 50% in all treated mice after long-term treatment by week 3 or 4 (Figure 4C). Importantly, FKBP12^F36V^-KRAS^G12V^ degradation upon dTAG^V^-1 administration dramatically increased the survival of tumor-bearing mice (Figure 4D). These results indicate that KRAS^G12V^ degradation by dTAG^V^-1 substantially reduced tumor growth and prolonged survival in the *KRAS^G12V^*-driven lung cancer model.

To extend these findings, we sought to validate the in vivo antitumor effects of KRAS^G12V^ degradation in pancreatic cancer. To do so, we utilized an isogenic pancreatic ductal adenocarcinoma cell line (PATU-8902 FKBP12^F36V^-KRAS^G12V^; *KRAS^–/–^*) that we previously developed to study KRAS^G12V^ degradation in vitro (29). We injected these cells s.c. into the flanks of nude mice. Once the tumor volume reached approximately 100 mm^3^, mice were randomized to either vehicle or dTAG^V^-1 treatment. Consistent with the results in our lung cancer GEMM, degradation of KRAS^G12V^ upon administration of dTAG^V^-1 significantly inhibited tumor growth of PATU-8902 FKBP12^F36V^-KRAS^G12V^; *KRAS^–/–^* cells (Figure 4, E and F). Collectively, these findings validate the efficacy of KRAS^G12V^ degradation across different types of cancer and support targeted degradation as an effective therapeutic strategy.

### KRAS^G12V^ degradation drives antitumor immunity through increased CD8^+^ T activity.

**Figure 5:**
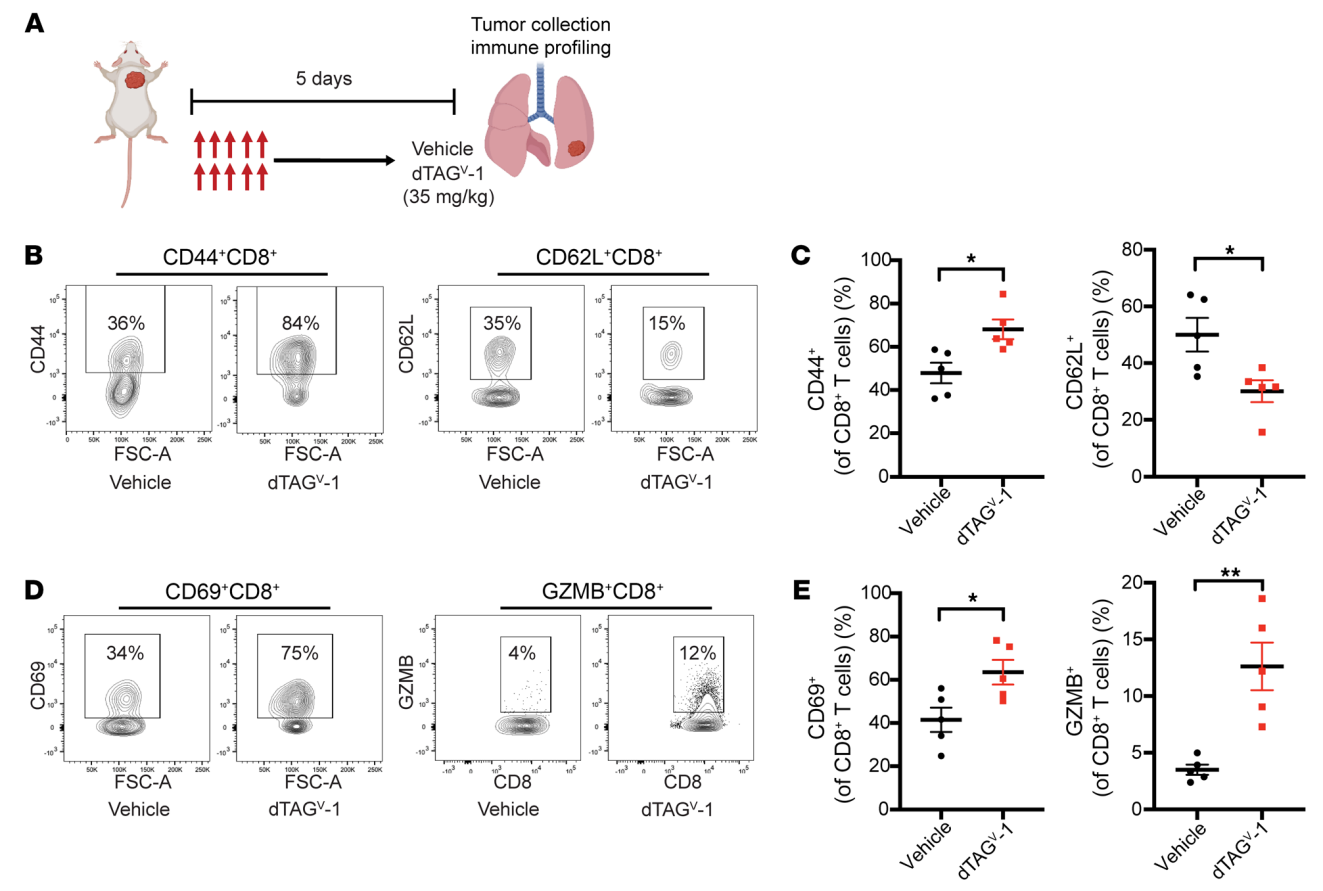
KRAS^G12V^ degradation increases CD8^+^ T activity in a *KRAS^G12V^*-driven lung cancer GEMM. (**A**) Schematic showing the experimental design for immune profiling. After confirming tumor burden by MRI, mice were randomized and treated once daily with either vehicle or dTAG^V^-1 (35 mg/kg) for 5 days. Tumor nodules were then collected, and tumor-infiltrating lymphocytes were analyzed by flow cytometry. The schematic was created using BioRender.com. (**B** and **C**) Frequencies of CD44^+^CD8^+^ and CD62L^+^CD8^+^ T cells (*n* = 5 per group). Data are presented as the mean ± SEM (**C**). (**D** and **E**) Frequencies of CD69^+^CD8^+^ and GZMB^+^CD8^+^ T cells (*n* = 5 per group). Data are presented as the mean ± SEM (**E**). *P < 0.05 and **P < 0.01, by 2-tailed Student’s *t* test (**C** and **E**).

Previous research has shown that KRAS inhibitors (AMG-510 and MRTX849) induce a proinflammatory TME and achieve durable responses alone or in combination with immune checkpoint inhibitors in preclinical mouse models (6, 45, 46). To investigate the immune-stimulatory effects of targeted degradation of KRAS^G12V^ in vivo, we profiled phenotypic and functional alterations of CD8^+^ T cells following a 5-day treatment with either vehicle or dTAG^V^-1 in tumor-bearing mice (Figure 5A). T cells with high CD44 expression (effector/memory marker) are characterized as effector cells, whereas T cells with high CD62L (naive T cell marker) are characterized as naive cells. Profiling of CD8^+^ T cells showed an increase in CD44^hi^ effector CD8^+^ T cells and a decrease in CD62L^hi^ naive CD8^+^ T cells upon KRAS^G12V^ degradation (Figure 5, B and C). To further assess the activation of CD8^+^ T cells, we analyzed the expression of an activation/costimulatory marker, CD69. KRAS^G12V^ degradation led to significantly higher frequencies of CD69^+^CD8^+^ T cells (Figure 5, D and E). Additionally, we evaluated the activity of cytotoxic T lymphocytes (CTLs) by staining for granzyme B (GzmB), a cytotoxic granule protein secreted by CD8^+^ T cells. An increase in GzmB^+^CD8^+^ T cells was observed upon FKBP12^F36V^-KRAS^G12V^ degradation, suggesting an enhanced cytotoxic T cell–mediated clearance of tumor cells (Figure 5, D and E). These findings suggest that KRAS^G12V^ degradation stimulates a robust antitumor immune program, potentially driven by activated CD8^+^ T cells.

### Transcriptomics analysis reveals that KRAS^G12V^ degradation triggers immune response signaling.

To explore how KRAS^G12V^ degradation affects immune response signaling in vivo, we performed a transcriptomics analysis of tumor nodules from mice treated with either vehicle or dTAG^V^-1 for 5 days (Supplemental Figure 3A). Gene set enrichment analysis (GSEA) of differentially expressed genes (dTAG^V^-1 versus vehicle) identified the most modulated pathways (Supplemental Figure 3B). FKBP12^F36V^-KRAS^G12V^ degradation led to the downregulation of genes associated with the cell cycle (Supplemental Figure 3, B–D), E2F targets (Supplemental Figure 3, E and F), and mitosis (Supplemental Figure 3, G and H). Notably, FKBP12^F36V^-KRAS^G12V^ degradation led to the upregulation of pathway genes associated with the inflammatory response, the IFN-γ response, the IFN-α response, and allograft rejection (Supplemental Figure 3, I–L). Heatmaps for the most differentially regulated genes in these top signatures induced upon FKBP12^F36V^-KRAS^G12V^ degradation showed an increased expression of numerous central proinflammatory cytokines and chemokines, including *Tnf*, *Cxcl10* and *Ccl5* (Supplemental Figure 3M). These factors secreted in the TME can potentially contribute to an optimal antitumor T cell response. To confirm these findings, we then used quantitative reverse transcription PCR (qRT-PCR) to measure the expression of *CCL5*, *CXCL10*, and *TNF* upon dTAG^V^-1 treatment in AALE cells expressing FKBP12^F36V^-KRAS^G12V^. dTAG^V^-1 treatment significantly upregulated *CCL5* and *CXCL10* expression, with a trend toward increased *TNF* expression (Supplemental Figure 3N). Furthermore, our RNA-Seq data also demonstrated that FKBP12^F36V^-KRAS^G12V^ degradation increased the expression of numerous granzyme subfamily member genes, including *Gmza*, *Gzmb*, *Gzmc*, as well as *Prf1* and *Ifng* (Supplemental Figure 3M), which are crucial for CD8^+^ T cell–mediated cytotoxicity. These results, in line with the immune profiling data, support the immune-stimulatory effects of KRAS^G12V^ degradation.

### KRAS^G12V^ degradation reprograms the TME to enhance antitumor immunity.

**Figure 6:**
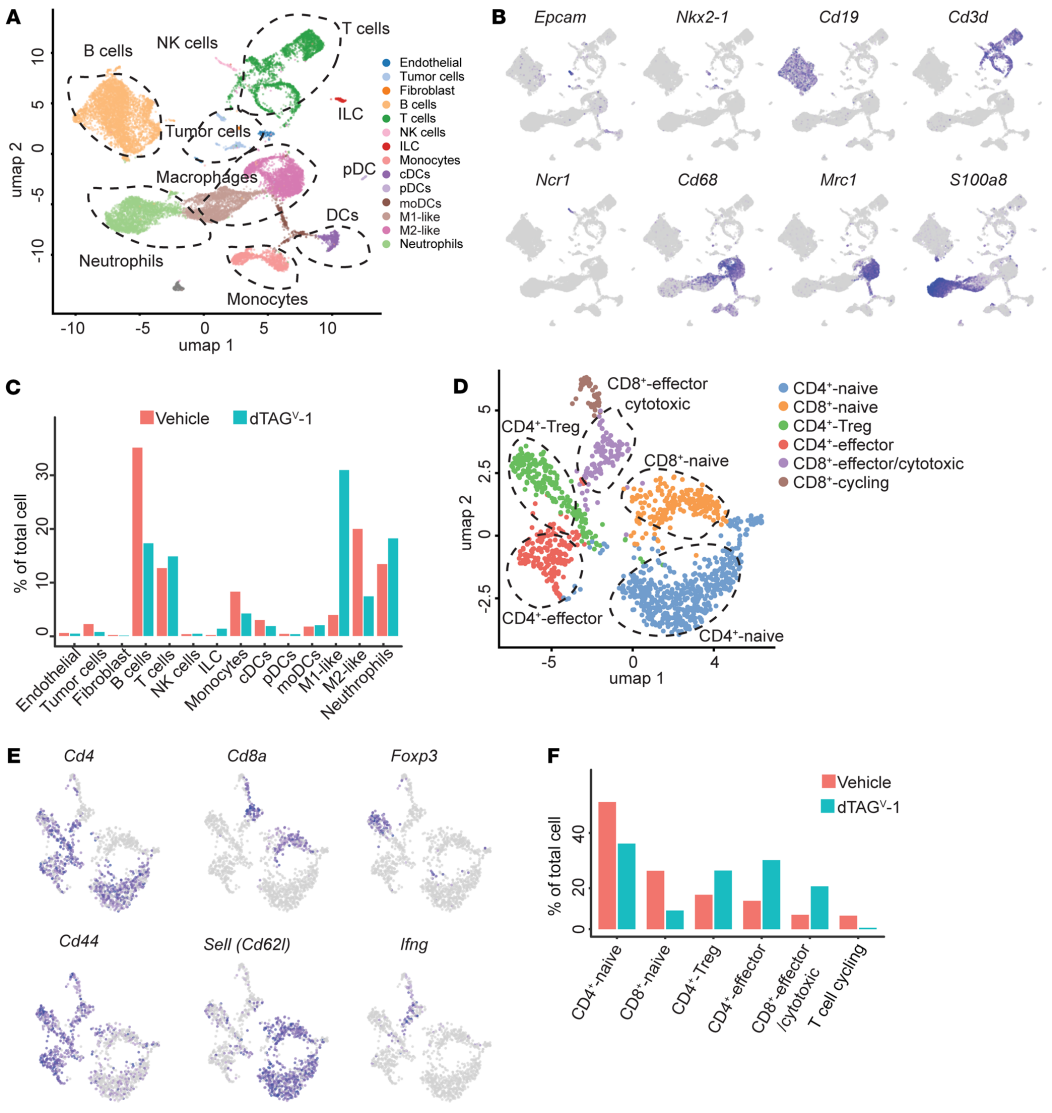
scRNA-Seq reveals that KRAS^G12V^ degradation reprograms the TME to promote antitumor immunity in a *KRAS^G12V^*-driven lung cancer GEMM. (**A**) Uniform manifold approximation and projection (UMAP) plot shows the identified cell populations including tumor cells, immune cells, and fibroblasts. (**B**) UMAP plots showing the expression of cell-type–specific marker genes. (**C**) Percentage of cells in the TME of annotated clusters in response to the indicated treatments. (**D**) UMAP plot shows the identified cell subsets in the T cell population. (**E**) UMAP plots show the expression of selected marker genes. (**F**) Percentage of cells in the annotated T cell subsets in response to the indicated treatments.

We next performed single-cell RNA-Seq (scRNA-Seq) to systematically examine the effect on the TME upon degradation of KRAS^G12V^. Lung tumors were collected after 5 days of treatment with either vehicle or dTAG^V^-1 to degrade FKBP12^F36V^-KRAS^G12V^ in tumor-bearing mice. We collected single-suspension cells and sequenced them on the 10X Genomics platform. In total, we obtained single-cell transcriptomes for 11,011 cells from the vehicle group and 7,486 cells from the dTAG^V^-1 cohort. Using unsupervised clustering, we identified approximately 14 distinct cell clusters according to the gene expression signatures (Figure 6A and Supplemental Figure 4A). We annotated these clusters with canonical cell-type markers and identified tumor cells expressing *Epcam* and *Nkx2-1*, B cells expressing *Cd19*, T cells expressing *Cd3d*, and NK cells expressing *Ncr1*. We also identified various myeloid cell populations, including monocytes, classical DCs (cDCs), plasmacytoid DCs (pDCs) (marked by *Siglech*, *Bst2*, and *Tlr7*), monocyte-derived DCs (marked by *Itgax*, *Flt3*, and *Mgl2*), macrophages (both M1-like and M2-like), and neutrophils (*S100a8*) (Figure 6B and Supplemental Figure 4A).

To dissect the TME alterations following KRAS^G12V^ degradation, we analyzed the immune cell subpopulations. In comparison with the vehicle-treated cohort, dTAG^V^-1 administration slightly increased the overall frequency of total immune cell populations (Supplemental Figure 4B). We noted a modest increase in the frequency of T cells, monocyte-derived DCs (moDCs), NK cells, as well as innate lymphoid cells (ILCs) upon FKBP12^F36V^-KRAS^G12V^ degradation (Figure 6C). Conversely, a decrease in the percentages of B cells and monocytes was observed upon FKBP12^F36V^-KRAS^G12V^ degradation (Figure 6C). Macrophages are broadly classified into 2 main phenotypes on the basis of their activation states: classically activated (M1) and alternatively activated (M2) (47, 48). While M1-like macrophages can exert antitumor effects, M2-like macrophages often contribute to tumor growth and immune evasion (47, 48). Consistent with previous studies in the murine and human lung tumors (49, 50), the macrophages in the lung TME largely belonged to the M2-like macrophage phenotype, expressing *Chil3* and *Mrc1* (Supplemental Figure 4A). Notably, our scRNA-Seq analysis revealed that dTAG^V^-1 treatment led to an increase in M1-like macrophages expressing *Ccl3*, *Tnf*, *Ler3*, *Clec4n*, *Tlr2/4*, and *Cd80* (51), whereas we observed a decrease in M2-like macrophages expressing *Chil3* and *Mrc1* (Figure 6C and Supplemental Figure 4A). The reduction in M2-like macrophages was further validated by IHC staining for MRC1 (also known as CD206) in the lung tumors upon FKBP12^F36V^-KRAS^G12V^ degradation (Supplemental Figure 4, C and D). These findings suggest that KRAS^G12V^ degradation might have an effect on promoting tumor-associated macrophages toward an M1-like antitumor phenotype. Given the high degree of heterogeneity and plasticity of macrophages, further investigation and functional validation are warranted in future work.

In addition, accumulating evidence indicates that B cells are strongly enriched in the TME in murine tumor models as well as in patients with lung cancer (49, 52–54). In agreement with this, our scRNA-Seq analysis revealed that B cells constituted a major immune cell population infiltrating the murine KRAS^G12V^ tumors. Unsupervised clustering of B cells identified 4 distinct clusters (Supplemental Figure 4E). Consistent with recent findings (52), most tumor-infiltrating B cells were in cluster 1, which exhibited a highly activated phenotype, expressing *Cd86* and *Cxcr4*. Cluster 2 B cells, expressing *Fcrl5*, had a memory-like phenotype (55, 56) (Supplemental Figure 4F). Cluster 3 B cells, expressing *Hspa1a*, *Hspa1b*, and *Jun*, were associated with an activated phenotype, whereas cells in cluster 4, which was the smallest group, showed high expression of *Iglc1* (Supplemental Figure 4F). Interestingly, FKBP12^F36V^-KRAS^G12V^ degradation led to a decrease in the percentage of cluster 1B cells compared with the vehicle group, whereas the frequency of cluster 3B cells increased (Supplemental Figure 4G). The percentages of clusters 2 and 4 remained similar upon FKBP12^F36V^-KRAS^G12V^ degradation. These observations suggest that KRAS^G12V^ degradation differentially affected various subtypes of activated B cells, which merits further investigation in the future.

Our in vivo immune-profiling analysis suggested that KRAS^G12V^ degradation increased CD8^+^ T cell activity in the TME. To comprehensively characterize the T cell subpopulations induced upon FKBP12^F36V^-KRAS^G12V^ degradation, we further analyzed the scRNA-Seq dataset and performed unbiased clustering of T cells. This approach identified 6 major clusters defined by the expression of marker genes, suggesting heterogeneous and complex populations. In the CD8^+^ T cell populations, cells with a high level of *Sell* and low levels of *Cd44* and *Ifng* expression were consistent with naive and inactivated states and were thus classified as a “CD8^+^-naive” cluster. FKBP12^F36V^-KRAS^G12V^ degradation reduced the percentage of naive CD8^+^ cells (Figure 6, D–F). Cells in clusters with high *Ifng* and *Cd44* expression resembled cytotoxic T cells and effector T cells, which were therefore classified as “CD8^+^ effector and cytotoxic T cells.” FKBP12^F36V^-KRAS^G12V^ degradation caused an increase in the effector and cytotoxic CD8^+^ T cells (Figure 6, D–F). Additionally, in the CD4^+^ T cell populations, we also observed a similar trend of decreased CD4^+^-naive T cells and increased CD4^+^ effector T cells (Figure 6, D–F). Our unbiased clustering also identified CD4^+^ Tregs, which expressed high levels of *Foxp3* (Figure 6, D–F). We detected an increase in the frequency of CD4^+^ Tregs upon FKBP12^F36V^-KRAS^G12V^ degradation, which might indicate potential feedback from increased effector and cytotoxic T cell activity.

**Figure 7:**
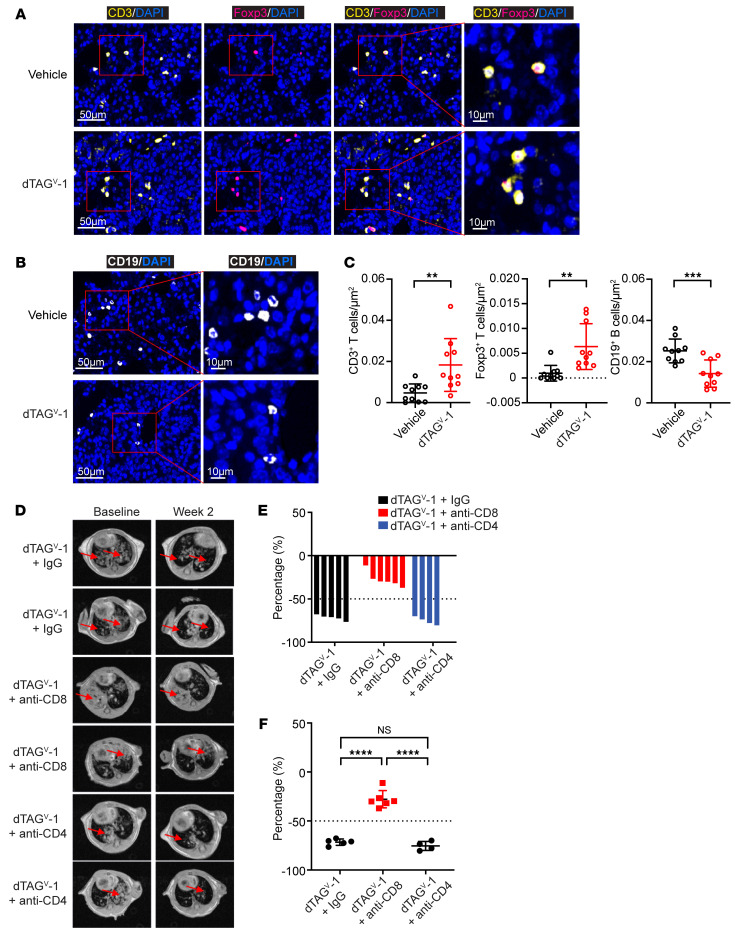
Antitumor immunity by KRAS^G12V^ degradation is partly dependent on CD8^+^ T cells in a *KRAS^G12V^*-driven lung cancer GEMM. (**A** and **B**) Representative multiplex IF images showing (**A**) tumor-infiltrating CD3^+^ T cells, Foxp3^+^ Tregs, and (**B**) CD19^+^ B cells in response to the indicated treatments. The same samples are presented in **A** and **B**. Scale bars: 50 μm and 10 μm (left to right, respectively). (**C**) Quantification of CD3^+^ T cells, Foxp3^+^ Tregs, and CD19^+^ B cells in response to the indicated treatments. Data are presented as the mean ± SD of 10 representative areas from 3 mice per group. (**D**) Representative MRI scans of lung tumors at baseline and 2 weeks in response to the indicated treatment. Red arrows indicate lung tumors. (**E** and **F**) Waterfall plot (**E**) and dot plot (**F**) showing changes in tumor volume compared with baseline after 2 weeks of treatment. Data are presented as the mean ± SD of 4–6 per group. **P < 0.01, ***P < 0.001, and ****P < 0.0001, by 2-tailed Student’s *t* test (**C**) and 1-way ANOVA with post hoc Tukey’s test (**F**).

To complement our scRNA-Seq findings of immune TME alterations, we performed multiplex immunofluorescence (IF) analysis of lung tumors from mice that were subjected to a 5-day treatment with either vehicle or dTAG^V^-1 (Figure 7, A–C). We consistently observed an increase in CD3^+^ T cells upon abrupt loss of FKBP12^F36V^-KRAS^G12V^ compared with vehicle treatment (Figure 7, A and C). Likewise, dTAG^V^-1 treatment also led to a higher percentage of Foxp3^+^ Tregs (Figure 7, A and C). In addition, similar to our observations using scRNA-Seq analysis, IF imaging showed that the frequency of CD19^+^ B cells was decreased upon FKBP12^F36V^-KRAS^G12V^ degradation (Figure 7, B and C).

In summary, in line with the in vivo immune-profiling and bulk transcriptomics analysis, our scRNA-Seq analysis, complemented with multiplex IF imaging, confirmed an antitumor immune response following dTAG^V^-1 treatment to degrade FKBP12^F36V^-KRAS^G12V^. These alterations included (a) a slight increase in overall immune cell infiltration, (b) a decrease in M2-like and an increase in M1-like macrophages, (c) a decrease in B cells, (d) a reduction in naive CD8^+^ and CD4^+^ T cells, and (e) an increase in effector and cytotoxic CD8^+^ T cells. These data further support the beneficial effects of targeted degradation of KRAS^G12V^ in rewiring the TME to enhance antitumor immunity.

### Antitumor immunity by KRAS^G12V^ degradation is partly dependent on CD8^+^ T cells.

Our integrated analysis above demonstrated that the antitumor immunity by KRAS^G12V^ degradation centered on T cells. To determine whether CD8^+^ or CD4^+^ T cells directly contribute to the antitumor response by dTAG^V^-1 treatment, we assessed the effect of perturbing immune cell function by in vivo neutralization antibodies against CD8 or CD4 (Supplemental Figure 5). FKBP12^F36V^-KRAS^G12V^ tumor–bearing mice were randomized to dTAG^V^-1 treatment or to a combination of dTAG^V^-1 with either anti-CD8 or anti-CD4 antibodies. Notably, compared with nondepletion of T cells in mice in the dTAG^V^-1–treated group, CD8^+^ T cell-depleted mice had significantly higher tumor burdens (Figure 7, D–F). Interestingly, we observed no significant difference between nondepletion mice and CD4^+^ T cell–ablated mice (Figure 7, D–F). These findings suggest that depletion of CD8^+^, but not CD4^+^, T cells mitigated the antitumor effect of FKBP12^F36V^-KRAS^G12V^ degradation by dTAG^V^-1, highlighting the observation that antitumor immunity by KRAS^G12V^ degradation was partly dependent on CD8^+^ T cells.

In summary, our work offers insights into how KRAS^G12V^ degradation influences both tumor progression and the immune response, underscoring the use of degraders as a potent strategy for targeting *KRAS*-mutant cancers. Furthermore, our study highlights the potential of the dTAG system in developing GEMMs for the study and validation of drug targets.

## Discussion

Targeted protein degradation holds incredible promise as a therapeutic strategy in diseases including cancer (14–17, 57). There is a current lack of targeted degradation strategies to study the consequences of drug target loss in vivo. Here, we focused on KRAS^G12V^, which is the second most common mutation in NSCLC and a driver in several cancers including pancreatic and colorectal cancer (58, 59). While breakthroughs in the development of KRAS^G12C^ inhibitors including AMG-510 (sotorasib) (9, 10, 60) and MRTX849 (adagrasib) (61) represent a paradigm shift in the clinical management of patients with NSCLC harboring a *KRAS^G12C^* mutation, there is currently a lack of selective *KRAS^G12V^* inhibitors. As the field moves toward targeting other additional KRAS mutants, an improved understanding of targeting KRAS^G12V^ in vivo is necessary. We aimed to advance the dTAG system to generate a degradable cancer GEMM using KRAS^G12V^ as a prioritized target.

In this study, we demonstrate that this mouse model harboring a tagged allele of *KRAS^G12V^* recapitulated the development of human adenocarcinoma. Our *FKBP12^F36V^-KRAS^G12V^* mouse strain developed lung adenocarcinoma with complete penetrance and a consistent latency period, comparable to previously reported *KRAS^G12V^* models (32–35). Critically, treatment with dTAG molecules effectively modeled the effect of targeted degradation of KRAS^G12V^. In the mice, dTAG^V^-1 administration led to robust and durable degradation of KRAS^G12V^, along with pronounced inhibition of downstream signaling, consistent with previous findings from studies using KRAS inhibitors in murine cancer models (6, 7). Strikingly, dTAG^V^-1 considerably reduced tumor growth in all treated *KRAS^G12V^* mice, and the FKBP12^F36V^ tag did not affect the kinetics of KRAS^G12V^ transformation or tumorigenesis in vitro or in vivo. Furthermore, although we focused on developing an NSCLC GEMM, our incorporation of *Cre*-recombinase–mediated introduction of FKBP12^F36V^-KRAS^G12V^ supports similar application in other tissues and cancers of interest including pancreatic cancer. Toward this aim, we performed experiments that extend to pancreatic cancer and demonstrate that dTAG^V^-1–mediated KRAS^G12V^ degradation drastically inhibited tumor growth in the PATU-8902 pancreatic cancer model. Our study demonstrates the unique power of these mouse models for in vivo evaluation of the effects of KRAS^G12V^ degradation on tumorigenesis.

Importantly, our GEMM enabled the evaluation of responses in an immune-competent mouse, which led us to test whether degradation of KRAS^G12V^ leads to an increased immune response in vivo. Prior work has linked KRAS^G12C^ inhibition to an immune response (46). In preclinical studies, treatment with AMG-510 showed a proinflammatory TME and induced durable cures alone and in combination with immune checkpoint inhibitors (6). Likewise, MRTX849 demonstrated an enhanced antitumor immunity, partly through increased MHC class I protein expression and decreased levels of immunosuppressive factors (45). MRTX849 also sensitized tumors to immune checkpoint inhibitors (45). Like these observations, we found that KRAS^G12V^ degradation drove antitumor immunity by increasing CD8^+^ T cell activity. This was further manifested by a substantial increase in CD44^hi^ effector CD8^+^ T cells, as well as CD69^+^CD8^+^ and GzmB^+^CD8^+^ cytotoxic T cells. Complementing these immune-profiling data, our transcriptomics analysis revealed that KRAS^G12V^ degradation caused a strong inhibition of KRAS-dependent downstream signaling (E2F, mitosis, and cell-cycle pathways) while also triggering robust immune response signaling.

Given our limited understanding of how KRAS^G12V^ affects the lung TME, we conducted scRNA-Seq analysis to identify global alterations in the TME following KRAS^G12V^ degradation. This analysis was complemented with further IHC and multiplex imaging staining. Our study uncovered several key observations and mechanisms of action on immune components. KRAS^G12V^ degradation upon dTAG^V^-1 administration (a) triggered the expansion and reduction of certain subtypes of tumor-infiltrating lymphoid (T and B cells) and myeloid cells (M1-like and M2-like macrophages and DCs), (b) promoted a shift of naive CD4^+^ and CD8^+^ T cells to effector/activated T cells and cytotoxic CD8^+^ T cells, and (c) elicited an increase in the expression of an antitumor cytotoxic gene signature. Supporting this, our in vivo T cell depletion assays showed that a functional immune system centered on CD8^+^ T cells was required for the antitumor response induced upon KRAS^G12V^ degradation. Additionally, our results also indicate that KRAS^G12V^ degradation may promote tumor-associated macrophages toward a M1-like antitumor phenotype and affect different subtypes of B cells, which merits further investigation. Notably, there is emerging interest in utilizing covalently modified peptide–MHC class I complexes as tumor-specific neoantigens with KRAS^G12C^ inhibitors (62, 63). Future work is necessary to determine whether KRAS degradation promotes the production of neoantigen peptides and whether this phenomenon contributes to antitumor immunity. This research will also help experimentally rule out the possibility of an FKBP12^F36V^ tag–induced immune response.

Our study also expands the use of the dTAG system for in vivo modeling. We and others have shown that the dTAG system can be used in xenograft models (27, 28, 37, 40, 64, 65) and mouse models of embryonic development (38). An important consideration in these efforts is to ensure that the tagged protein is functional and maintains the expected level of expression. One limitation of tag-based systems is that the addition of a tag has the potential to alter protein stability and half-life (66). In GEMMs, endogenous fusion with the FKBP12^F36V^-tag may affect protein stability and half-life, decreasing protein expression in vivo (39, 67). In our oncogene induction model, *FKBP12^F36V^-KRAS^G12V^* is driven by a PGK promoter. Studies in embryonic development suggest that this may be target specific (38), but future work is warranted to improve tagging strategies to maintain protein stability to address this limitation. Furthermore, our oncogene induction model does not allow for the evaluation of the tolerability of systemic KRAS degradation. GEMMs that utilize the dTAG system, such as those recently described for CDK2 and CDK5, will prove to be highly complementary for evaluating toxicities from specific target protein loss (39).

In line with other studies, this work provides preclinical evidence that targeted degradation is a powerful strategy to disrupt mutant KRAS in vivo (21–25, 68). Recently, a clinical KRAS^G12D^ degrader (ASP3082) was described to have potent antitumor activities in multiple *G12D*-driven mouse models of cancer including pancreatic, colorectal, and NSCLC cancer (26). Currently, a phase I clinical trial is underway in patients with previously treated, locally advanced, or metastatic solid tumors with the *KRAS^G12D^* mutation (NCT05382559). While it remains an open question whether KRAS degradation will provide a benefit over inhibition, our work highlights the therapeutic potential of targeted degradation of KRAS. It is worth noting that because of the current unavailability of KRAS^G12V^-specific inhibitors, a direct comparison of the immunological effects between degradation of KRAS^G12V^ and inhibition of KRAS^G12V^ is not yet experimentally achievable. When these inhibitors become available, our mouse model will serve as an important platform for evaluating the differential effects on downstream signaling, tumorigenesis, and TME alterations, allowing for a comprehensive comparison of the responses to inhibitors and degraders. With the emergence of pan-KRAS and RAS(ON) multi-selective inhibitors (69–72), it will also be interesting to evaluate the immune responses triggered by these inhibitors and dTAG^V^-1–mediated degradation in our model.

Interestingly, a recent study showed that *Kras* oncogene ablation could prevent resistance to KRAS inhibitors in advanced lung adenocarcinomas, supporting the potential benefit of protein degradation (34). Supported by our prior cellular studies using the dTAG system studying targeted agent resistance mechanisms (28, 73), we expect that our model will enable the identification of mechanisms of resistance to KRAS disruption and the testing of drug combination strategies in vivo. Future work will be necessary to evaluate drug combination approaches and to extend our model to additional *KRAS* mutants and other *KRAS*-driven cancers. In summary, our study demonstrates that degradation of KRAS^G12V^ drove antitumor immunity and abolished tumor growth in lung cancer. Our work highlights the value of degradable model systems to understand and advance targeted degradation strategies for cancer therapy.

## Methods

### Sex as a biological variable.

Our study examined male and female animals, and similar findings are reported for both sexes.

### Generation of FKBP12^F36V^-KRAS^G12V^ transgenic mice.

To generate FKBP12^F36V^-KRAS^G12V^ mice that enable specific degradation by dTAG^V^-1, we designed a targeting vector with a PGK promoter and a Lox-Stop-Lox cassette, which allows the temporal and spatial control of gene expression, as we previously described (43). *FKBP12^F36V^-KRAS^G12V^* complementary DNA was cloned into the targeting vector. Transgene expression is controlled by the stop cassette, which can be removed by Cre recombinase. After the targeting vector was electroporated into ES cells, these cells were engineered to allow single-copy transgene insertion at the *Co1lA1* locus. ES clones that carry the *FKBP12^F36V^-KRAS^G12V^* transgene were selected, expanded, and used to inject into B6 blastocysts, which gave rise to chimeras. The chimeras were bred with WT B6 mice, and transgene-positive mice were genotyped and sequenced and expanded for experiments. From 6–8 weeks of age, mice were given adenovirus-SPC-Cre recombinase (Ad-Cre) by intranasal intubation to allow Cre-Lox–mediated recombination.

### In vivo studies.

For NIH/3T3 KRAS^G12V^ or FKBP12^F36V^-KRAS^G12V^ mouse model studies, 1 × 10^6^ cells were injected into the flank of nude mice. Tumor growth was monitored and measured by caliper. For treatment studies using *FKBP12^F36V^-KRAS^G12V^* GEMMs, mice were evaluated by MRI (Preclinical Imaging Laboratory, NYU Grossman School of Medicine and In Vivo Imaging Facility, University of Pittsburgh UPMC Hillman Cancer Center) to quantify lung tumor burden before randomization and after drug treatment. Once the tumor volumes reached approximately 100 mm^3^ in size (quantified by a 3D slicer using MRI images), the mice were then enrolled and randomized into either the vehicle or dTAG^V^-1 (35 mg/kg) treatment group. For the treatment studies using the PATU-8902 pancreatic cancer model, 1 × 10^6^ cells were injected into the flank of nude mice. Tumor volumes were monitored and measured by caliper before randomization. Once tumor volumes reached approximately 100 mm^3^ in size, mice were randomized to treatment with either vehicle or dTAG^V^-1 (35 mg/kg). For CD8^+^ or CD4^+^ T cell depletion studies, mice were injected i.p. with either anti-CD8 antibody (400 mg, Bio X Cell, clone 2.43), anti-CD4 (400 mg, Bio X Cell, clone GK1.5), or an isotype control antibody 48 and 24 hours before beginning dTAG^V^-1 treatment and then every 4 days thereafter.

### Extended material and methods.

Additional details on compounds, reagents, assays, and bioinformatics analysis are provided in the Supplemental Methods.

### Statistics.

Statistical analyses were performed using GraphPad Prism, version 10 (GraphPad Software), and statistical significance was determined if the *P* value was less than 0.05. Data are presented as the mean with SD unless otherwise specified. Statistical comparisons of 2 groups were performed using a 2-tailed Student’s *t* test, and multiple comparisons were performed using 1-way ANOVA followed by post hoc Dunnett’s test or Tukey’s test unless otherwise specified.

### Study approval.

All animal studies were reviewed and approved by the IACUC of the NYU Grossman School of Medicine and the University of Pittsburgh School of Medicine. Both male and female mice were used, and all mice were maintained in accordance with guidelines of the NYU Grossman School of Medicine and the University of Pittsburgh School of Medicine with regard to the care, welfare, and treatment of laboratory animals. All experiments met or exceeded the standards of the Association for the Assessment and Accreditation of Laboratory Animal Care, International (AALAC), the US Department of Health and Human Services, and all local and federal animal welfare laws.

### Data availability.

The the raw and processed bulk RNA-Seq and scRNA-Seq data generated and reported in this work are available in the NCBI’s Gene Expression Omnibus (GEO) database (GEO GSE234472). All supporting data are provided in the Supporting Data Values file and are available online as supplemental material.

## Author contributions

HZ, KKW, and BN conceptualized the study, designed the experiments, interpreted the data, wrote the manuscript, and supervised the study. DL, KG, JG, and SK performed most of the experiments, analyzed and interpreted the data, and contributed to the writing of the manuscript. YH performed bioinformatics analyses. ATO, CCK, YP, FS, HMK, AMW, Y Li, TC, CT, MR, MM, DSL, JC, MSDP, JS, AC, JMP, XS, ZYZ, and MP conducted experiments, including in vitro assays in NIH/3T3 and AALE cells, multiplex imaging, MRI scans, dosing, and IHC. FL, HH, SSG, TZ, BH, BAN, WC, MEK, XW, JL, AHB, Y Liu, XZ, TCB, and NSG provided resources and analyzed and interpreted the data. The order in which the co-first authors are listed was determined by the order of their entry into the study. All authors reviewed the manuscript.

## Supplementary Material

Supplemental data

Unedited blot and gel images

Supporting data values

## Version 1 12/24/2024

In-Press Preview

## Version 2 01/16/2025

Electronic publication

## References

[1] Siegel RL (2019). Cancer statistics, 2019. CA Cancer J Clin.

[2] Cheng L (2012). Molecular pathology of lung cancer: key to personalized medicine. Mod Pathol.

[3] Scheffler M (2019). K-ras mutation subtypes in NSCLC and associated co-occuring mutations in other oncogenic pathways. J Thorac Oncol.

[4] Janes MR (2018). Targeting KRAS mutant cancers with a covalent G12C-specific inhibitor. Cell.

[5] Lanman BA (2020). Discovery of a covalent inhibitor of KRAS^G12C^ (AMG 510) for the treatment of solid tumors. J Med Chem.

[6] Canon J (2019). The clinical KRAS(G12C) inhibitor AMG 510 drives anti-tumour immunity. Nature.

[7] Hallin J (2020). The KRAS^G12C^ inhibitor MRTX849 provides insight toward therapeutic susceptibility of KRAS-mutant cancers in mouse models and patients. Cancer Discov.

[8] Jänne PA (2020). KRYSTAL-1: activity and safety of adagrasib (MRTX849) in advanced/metastatic non-small cell lung cancer (NSCLC) harboring KRAS G12C mutation. Eur J Cancer.

[9] Hong DS (2020). KRAS^G12C^ inhibition with sotorasib in advanced solid tumors. N Engl J Med.

[10] De Langen AJ (2023). Sotorasib versus docetaxel for previously treated non-small-cell lung cancer with KRAS^G12C^ mutation: a randomised, open-label, phase 3 trial. Lancet.

[11] Zhao Y (2021). Diverse alterations associated with resistance to KRAS(G12C) inhibition. Nature.

[12] Xue JY (2020). Rapid non-uniform adaptation to conformation-specific KRAS(G12C) inhibition. Nature.

[13] Kim D (2020). Targeting KRAS(G12C): from inhibitory mechanism to modulation of antitumor effects in patients. Cell.

[14] Sakamoto KM (2001). Protacs: chimeric molecules that target proteins to the Skp1-Cullin-F box complex for ubiquitination and degradation. Proc Natl Acad Sci U S A.

[15] Burslem GM, Crews C M (2020). Proteolysis-targeting chimeras as therapeutics and tools for biological discovery. Cell.

[16] Bekes M (2022). PROTAC targeted protein degraders: the past is prologue. Nat Rev Drug Discov.

[17] Chirnomas D (2023). Protein degraders enter the clinic - a new approach to cancer therapy. Nat Rev Clin Oncol.

[18] Bondeson DP (2015). Catalytic in vivo protein knockdown by small-molecule PROTACs. Nat Chem Biol.

[19] Burslem GM (2018). The advantages of targeted protein degradation over inhibition: an RTK case study. Cell Chem Biol.

[20] Koide E (2023). Development and characterization of selective FAK inhibitors and PROTACs with in vivo activity. Chembiochem.

[21] Zeng M (2020). Exploring targeted degradation strategy for oncogenic KRAS^G12C^. Cell Chem Biol.

[22] Bond MJ (2020). Targeted degradation of oncogenic KRAS^G12C^ by VHL-recruiting PROTACs. ACS Cent Sci.

[23] Popow J (2024). Targeting cancer with small-molecule pan-KRAS degraders. Science.

[24] Bery N (2020). A potent KRAS macromolecule degrader specifically targeting tumours with mutant KRAS. Nat Commun.

[25] Yang J (2024). A pan-KRAS degrader for the treatment of KRAS-mutant cancers. Cell Discov.

[26] Nagashima T (2023). Abstract 5735: novel KRAS G12D degrader ASP3082 demonstrates in vivo, dose-dependent KRAS degradation, KRAS pathway inhibition, and antitumor efficacy in multiple KRAS G12D-mutated cancer models. Cancer Res.

[27] Nabet B (2018). The dTAG system for immediate and target-specific protein degradation. Nat Chem Biol.

[28] Nabet B (2020). Rapid and direct control of target protein levels with VHL-recruiting dTAG molecules. Nat Commun.

[29] Ferguson FM (2020). Discovery of a selective inhibitor of doublecortin like kinase 1. Nat Chem Biol.

[30] Nabet B (2021). Charting a new path towards degrading every protein. Chembiochem.

[32] Guerra C (2003). Tumor induction by an endogenous K-ras oncogene is highly dependent on cellular context. Cancer Cell.

[33] Sanclemente M (2018). c-RAF ablation induces regression of advanced Kras/Trp53 mutant lung adenocarcinomas by a mechanism independent of MAPK signaling. Cancer Cell.

[34] Salmon M (2023). Kras oncogene ablation prevents resistance in advanced lung adenocarcinomas. J Clin Invest.

[35] Drosten M (2018). Genetically engineered mouse models of K-Ras-driven lung and pancreatic tumors: validation of therapeutic targets. Cold Spring Harb Perspect Med.

[36] Jaeger MG, Winter G E (2021). Fast-acting chemical tools to delineate causality in transcriptional control. Mol Cell.

[37] Bilal F (2021). The transcription factor SLUG uncouples pancreatic cancer progression from the RAF-MEK1/2-ERK1/2 pathway. Cancer Res.

[38] Abuhashem A (2022). Rapid and efficient degradation of endogenous proteins in vivo identifies stage-specific roles of RNA Pol II pausing in mammalian development. Dev Cell.

[39] Yenerall P (2023). Use of the dTAG system in vivo to degrade CDK2 and CDK5 in adult mice and explore potential safety liabilities. Toxicol Sci.

[40] Lin S (2022). An in vivo CRISPR screening platform for prioritizing therapeutic targets in AML. Cancer Discov.

[41] Vichas A (2021). Integrative oncogene-dependency mapping identifies RIT1 vulnerabilities and synergies in lung cancer. Nat Commun.

[42] Lo A (2021). Multiomic characterization of oncogenic signaling mediated by wild-type and mutant RIT1. Sci Signal.

[43] Akbay EA (2017). Interleukin-17A promotes lung tumor progression through neutrophil attraction to tumor sites and mediating resistance to PD-1 blockade. J Thorac Oncol.

[44] Jackson EL (2001). Analysis of lung tumor initiation and progression using conditional expression of oncogenic K-ras. Genes Dev.

[45] Briere DM (2021). The KRAS^G12C^ inhibitor MRTX849 reconditions the tumor immune microenvironment and sensitizes tumors to checkpoint inhibitor therapy. Mol Cancer Ther.

[46] Molina-Arcas M, Downward J (2024). Exploiting the therapeutic implications of KRAS inhibition on tumor immunity. Cancer Cell.

[47] Pan Y (2020). Tumor-associated macrophages in tumor immunity. Front Immunol.

[48] Wang S (2024). Targeting M2-like tumor-associated macrophages is a potential therapeutic approach to overcome antitumor drug resistance. NPJ Precis Oncol.

[49] Chen J (2020). Single-cell transcriptome and antigen-immunoglobin analysis reveals the diversity of B cells in non-small cell lung cancer. Genome Biol.

[50] Zilionis R (2019). Single-cell transcriptomics of human and mouse lung cancers reveals conserved myeloid populations across individuals and species. Immunity.

[51] Jablonski KA (2015). Novel markers to delineate murine M1 and M2 macrophages. PLoS One.

[52] Bod L (2023). B-cell-specific checkpoint molecules that regulate anti-tumour immunity. Nature.

[53] Morgan D, Tergaonkar V (2022). Unraveling B cell trajectories at single cell resolution. Trends Immunol.

[54] Lambrechts D (2018). Phenotype molding of stromal cells in the lung tumor microenvironment. Nat Med.

[55] Li H (2016). Fc receptor-like 5 expression distinguishes two distinct subsets of human circulating tissue-like memory B cells. J Immunol.

[56] Kim CC (2019). FCRL5^+^ memory B cells exhibit robust recall responses. Cell Rep.

[57] Paiva SL, Crews C M (2019). Targeted protein degradation: elements of PROTAC design. Curr Opin Chem Biol.

[58] Lee JK (2022). Comprehensive pan-cancer genomic landscape of KRAS altered cancers and real-world outcomes in solid tumors. NPJ Precis Oncol.

[59] Fernandez Montes A (2023). The frequency of specific KRAS mutations, and their impact on treatment choice and survival, in patients with metastatic colorectal cancer. Oncologist.

[60] Skoulidis F (2021). Sotorasib for lung cancers with KRAS p.G12C mutation. N Engl J Med.

[61] Janne PA (2022). Adagrasib in non-small-cell lung cancer harboring a KRAS^G12C^ mutation. N Engl J Med.

[62] Zhang Z (2022). A covalent inhibitor of K-Ras(G12C) induces MHC class I presentation of haptenated peptide neoepitopes targetable by immunotherapy. Cancer Cell.

[63] Hattori T (2023). Creating MHC-restricted neoantigens with covalent inhibitors that can be targeted by immune therapy. Cancer Discov.

[64] Nirala BK (2023). MYC regulates CSF1 expression via microRNA 17/20a to modulate tumor-associated macrophages in osteosarcoma. JCI Insight.

[65] Radko-Juettner S (2024). Targeting DCAF5 suppresses SMARCB1-mutant cancer by stabilizing SWI/SNF. Nature.

[66] Vetma V (2024). Confounding factors in targeted degradation of short-lived proteins. ACS Chem Biol.

[67] Mehta S (2022). Temporal resolution of gene derepression and proteome changes upon PROTAC-mediated degradation of BCL11A protein in erythroid cells. Cell Chem Biol.

[68] Yang F (2022). Efficient targeted oncogenic KRA^SG12^C degradation via first reversible-covalent PROTAC. Eur J Med Chem.

[69] Kim D (2023). Pan-KRAS inhibitor disables oncogenic signalling and tumour growth. Nature.

[70] Holderfield M (2024). Concurrent inhibition of oncogenic and wild-type RAS-GTP for cancer therapy. Nature.

[71] Wasko UN (2024). Tumour-selective activity of RAS-GTP inhibition in pancreatic cancer. Nature.

[72] Schulze CJ (2023). Chemical remodeling of a cellular chaperone to target the active state of mutant KRAS. Science.

[73] Sulahian R (2019). Synthetic lethal interaction of SHOC2 depletion with MEK inhibition in RAS-driven cancers. Cell Rep.

