# Supplementary material for: Targeted degradation of oncogenic KRASG12V triggers antitumor immunity in lung cancer models
Source: J Clin Invest. 2024 Dec 24;135(2):e174249. doi: 10.1172/JCI174249 (PMC11735103; doi:10.1172/JCI174249)
Supplement: Supplemental data [file jci-135-174249-s063.pdf]

Supplemental data

Targeted degradation of oncogenic KRAS^{G12V} triggers antitumor immunity in lung cancer models

Dezhi Li[1,19,#], Ke Geng[1,#], Yuan Hao[2,#], Jiajia Gu[3,#], Saurav Kumar[4,#], Annabel T. Olson[4], Christina C. Kuismi[4], Hye Mi Kim[3,5], Yuanwang Pan[1], Fiona Sherman[1], Asia M. Williams[3,5], Yiting Li[3,6], Fei Li[7,8], Ting Chen[1], Cassandra Thakurdin[1], Michela Ranieri[1], Mary Meynardie[1], Daniel S. Levin[1], Janaye Stephens[1], Alison Chafitz[1], Joy Chen[4], Mia S. Donald-Paladino[4], Jaylen M. Powell[1], Ze-Yan Zhang[9], ,Wei Chen[10], Magdalena Ploszaj[1], Han Han[1], Shengqing Stan Gu[11], Tinghu Zhang[12], Baoli Hu[3,13], Benjamin A. Nacev[3,14,15], Medard Ernest Kaiza[3,5], Alice H. Berger[4], Xuerui Wang[3,5], Jing Li[3,5], Xuejiao Sun[3], Yang Liu[16], Xiaoyang Zhang[17], Tullia C. Bruno[3,5], Nathanael S. Gray[12], Behnam Nabet[4,18*], Kwok-Kin Wong[1*], Hua Zhang[3,14*]

[1]Division of Hematology and Medical Oncology, Laura and Isaac Perlmutter Cancer Center, New York University Langone Health, New York, NY 10016, USA

[2]Applied Bioinformatics Laboratories, Office of Science and Research, New York University Grossman School of Medicine, New York, NY 10016, USA

[3]Hillman Cancer Center, UPMC, Pittsburgh, PA 15232 USA

[4]Human Biology Division, Fred Hutchinson Cancer Center, Seattle, WA 98109, USA

[5]Department of Immunology, University of Pittsburgh, Pittsburgh, PA 15261, USA

[6]School of Medicine, Tsinghua University, Beijing, China

[7]Department of Pathology, School of Basic Medical Sciences, Fudan University, Shanghai, China

[8]Frontier Innovation Center, School of Basic Medical Sciences, Fudan University, Shanghai, China

[9]Department of Radiation Oncology, New York University Grossman School of Medicine, New York, NY 10016, USA

[10]Division of Pulmonary Medicine, Department of Pediatrics, UPMC Children's Hospital of Pittsburgh and University of Pittsburgh, Pittsburgh, PA, USA

[11]Department of Hematopoietic Biology and Malignancy, University of Texas MD Anderson Cancer Center, Houston, TX, USA

[12]Department of Chemical and Systems Biology, Chem-H and Stanford Cancer Institute, Stanford School of Medicine, Stanford University, Stanford, CA 94305, USA

[13]Department of Neurological Surgery, University of Pittsburgh School of Medicine, Pittsburgh, PA 15261, USA

[14]Department of Medicine, Division of Hematology/Oncology, University of Pittsburgh School of Medicine, Pittsburgh, PA 15261, USA

[15]Department of Pathology, University of Pittsburgh School of Medicine, Pittsburgh, PA 15261, USA

[16]Department of Bioengineering, University of Illinois Urbana-Champaign, Urbana, IL 61801, USA

[17]Department of Oncological Sciences, Huntsman Cancer Institute, University of Utah, Salt Lake City, UT 84112, USA

[18]Department of Pharmacology, University of Washington, Seattle, WA 98195, USA

[19]Present address: Department of Pulmonary and Critical Care Medicine, Shandong Provincial Hospital Affiliated to Shandong First Medical University, Jinan, 250021, China

[#]DL, KG, YH, JG, and SK contributed equally to this article.

[*]**Corresponding Authors**: Hua Zhang, Hillman Cancer Center, UPMC, Department of Medicine, Division of Hematology/Oncology, University of Pittsburgh School of Medicine, 5117 Centre Avenue, Pittsburgh, PA 15261. Phone: 412-864-7742; Email: huz59@pitt.edu

Kwok-Kin Wong, Laura and Isaac Perlmutter Cancer Center, New York University Langone Medical Center, 550 First Avenue, New York, NY 10016, USA. Phone: 212-263-9203; Email: Kwok-Kin.Wong@nyulangone.org

Behnam Nabet, Human Biology Division, Fred Hutchinson Cancer Center, 1100 Fairview Avenue N., Seattle, WA 98109, USA. Phone: 206-667-4052; Email: bnabet@fredhutch.org

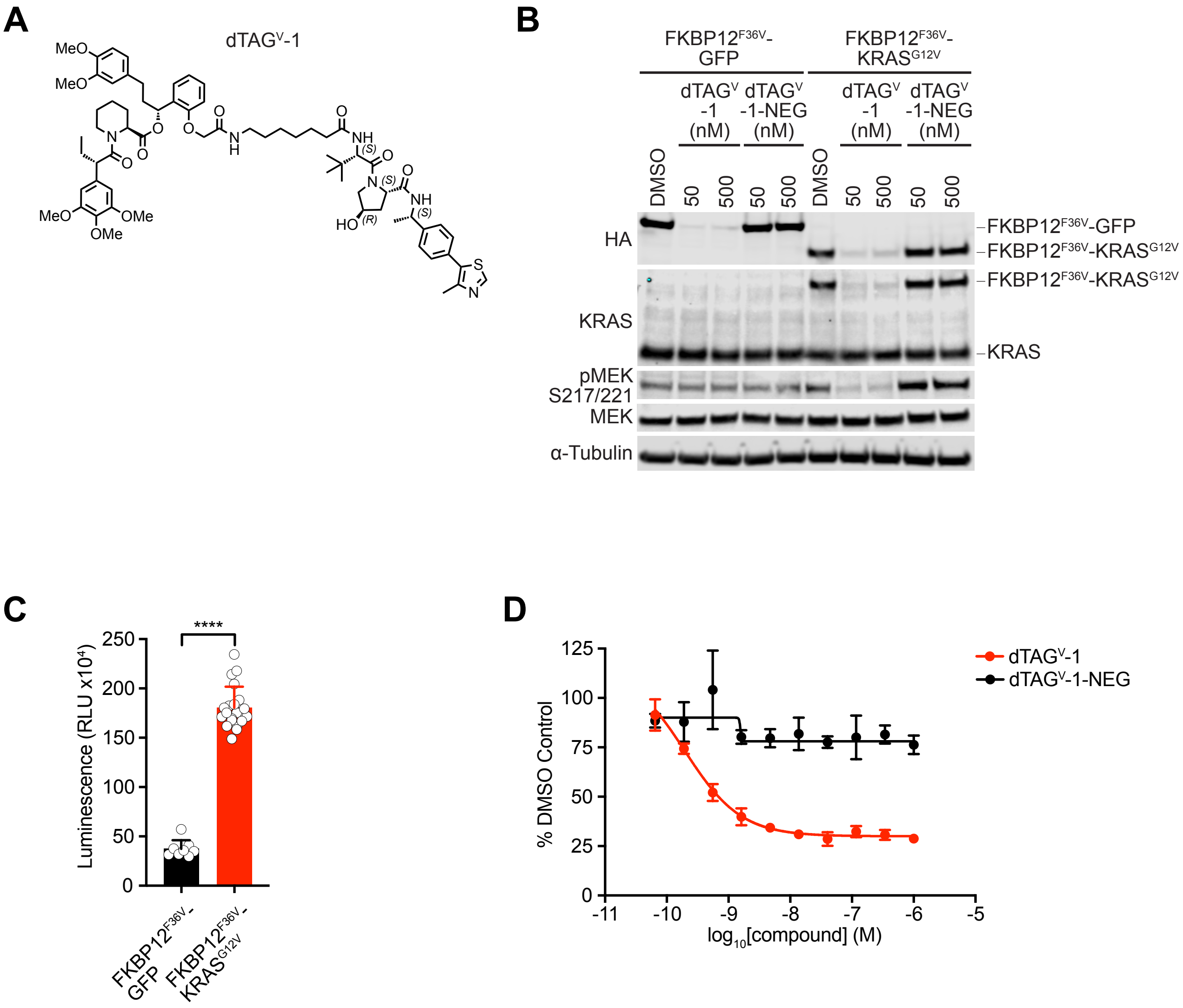

Supplemental Figure 1. Validation of targeted degradation of KRAS^{G12V} using the dTAG system.

(**A**) Chemical structure of dTAG^{V}-1. (**B**) Immunoblot analysis of HA to detect FKBP12^{F36V}-GFP or FKBP12^{F36V}-KRAS^{G12V}, KRAS, pMEK, MEK, and α-Tubulin of AALE cells expressing FKBP12^{F36V}-GFP or FKBP12^{F36V}-KRAS^{G12V} treated with DMSO, 50 or 500 nM dTAG^{V}-1, or 50 or 500 nM dTAG^{V}-1-NEG for 8 h. Data is representative of n = 3 independent experiments. (**C**) Antiproliferation of AALE cells expressing FKBP12^{F36V}-GFP or FKBP12^{F36V}-KRAS^{G12V} cultured as ultra-low adherent 3D-spheroid suspensions for 144 h. Data is presented as mean ± s.d. of n = 8 (FKBP12^{F36V}-GFP) or n = 21 (FKBP12^{F36V}-KRAS^{G12V}) biologically independent samples and are representative of n = 3 independent experiments. RLU = Relative light units. (**D**) DMSO-normalized antiproliferation of AALE cells expressing FKBP12^{F36V}-KRAS^{G12V} cultured as ultra-low adherent 3D-spheroid suspensions and treated with the indicated compounds for 120 h. Data is presented as mean ± s.d. of n = 4 biologically independent samples and are representative of n = 3 independent experiments. ****P < 0.0001 (**C**) by a two-tailed Student's t-test.

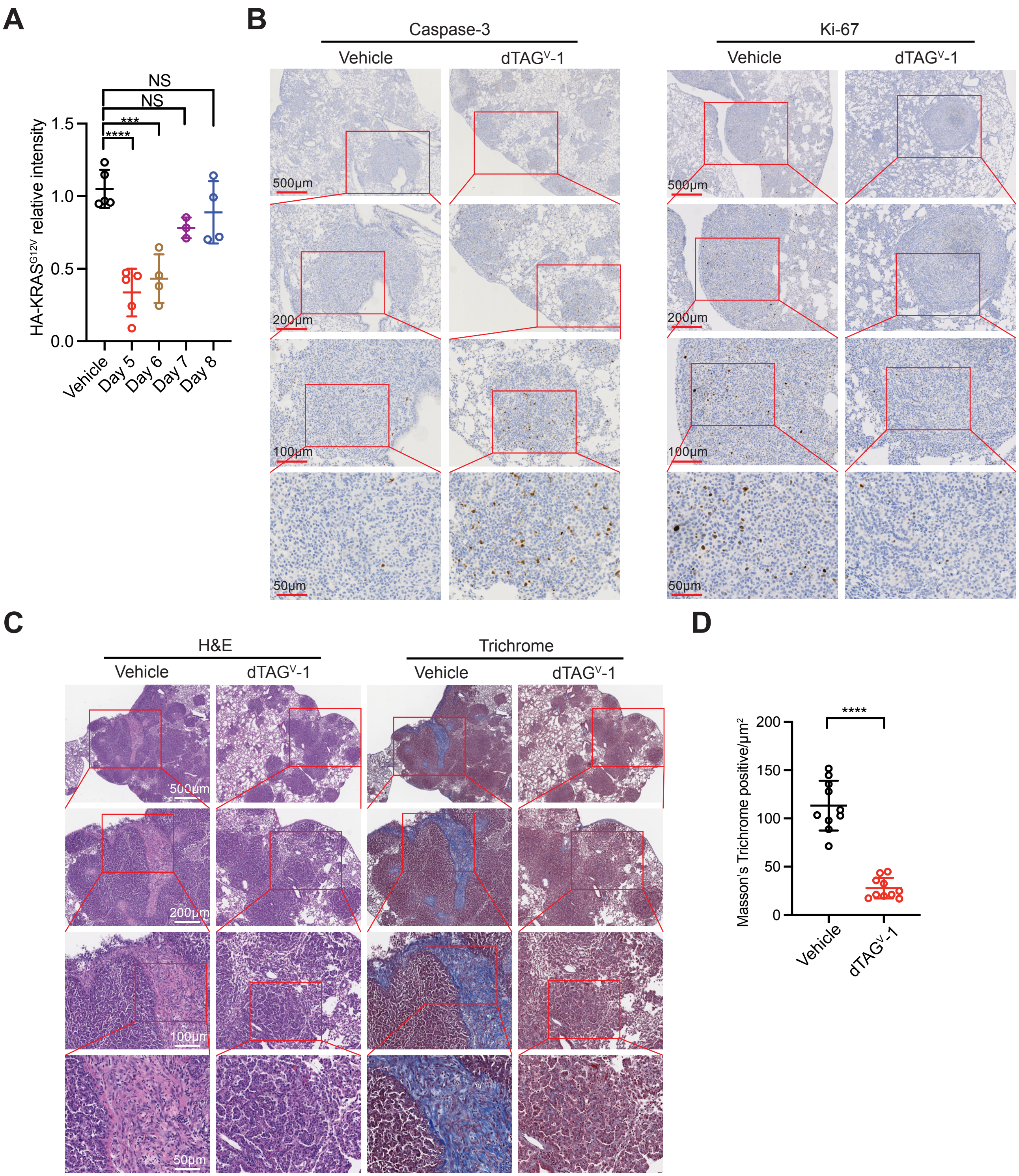

Supplemental Figure 2. dTAG^{V}-1 successfully degrades KRAS^{G12V} and confers anti-cancer effects in a *KRAS^{G12V}*-driven lung cancer GEMM.

(**A**) Quantification of HA to detect FKBP12^{F36V}-KRAS^{G12V} relative levels from immunoblot analysis in Figure 3B. Data is presented as mean ± s.d. from n = 3-5 per group. (**B**) Representative images of IHC staining for Ki-67 and cleaved caspase 3 of lung tumors after indicated treatment, related to Figure 3E. The scale bar represents 500, 200, 100 and 50 μm from top to bottom. (**C**) Representative images of H&E and Masson's trichrome staining of lung tumors after the indicated treatment. The scale bar represents 500, 200, 100 and 50 μm from top to bottom. (**D**) Quantification of Masson's trichrome staining after the indicated treatment. Data is presented as mean ± s.d. of ten representative areas from n = 3 mice per group. ***P < 0.001, ****P < 0.0001, non-significant (NS) by a one-way ANOVA with post hoc Dunnett's test (**A**) and a two-tailed Student's t-test (**D**).

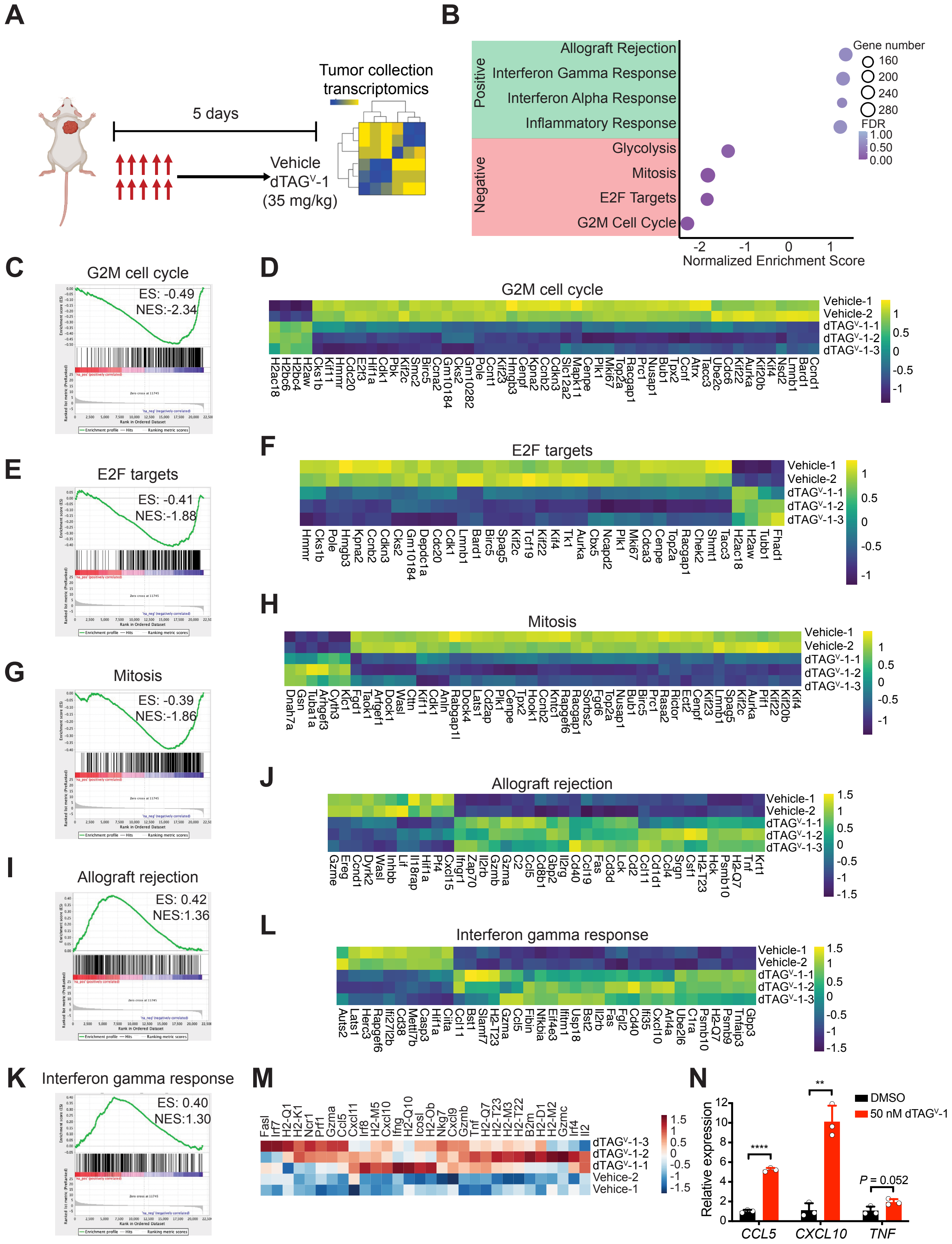

A
5 days
Tumor collection
transcriptomics
Vehicle
dTAGV-1
(35 mg/kg)
B
Positive
Allograft Rejection
Interferon Gamma Response
Interferon Alpha Response
Inflammatory Response
Negative
Glycolysis
Mitosis
E2F Targets
G2M Cell Cycle
-2 -1 0 1
Normalized Enrichment Score
Gene number
160
200
240
280
FDR
1.00
0.50
0.00
C
G2M cell cycle
ES: -0.49
NES:-2.34
D
G2M cell cycle
Vehicle-1
Vehicle-2
dTAGV-1-1
dTAGV-1-2
dTAGV-1-3
H2ac18 H2bc6 H2bc4 H2aw Cks1b Kif11 Hmmr Cdc20 E2f3 Hif1a Cdk1 Pbk Kif2c Smc2 Birc5 Ccna2 Gm10184 Cks2 Gm10282 Pole Ccnt1 Kif23 Hmgb3 Cenpf Kpna2 Ccnb2 Cdkn3 Slc12a2 Mapk11 Cenpe Plk1 Mki67 Top2a Racgap1 Prc1 Nusap1 Bub1 Tpx2 Ccnf Atrx Tacc3 Ube2c Cdc6 Kif22 Aurka Kif20b Kif4 Nsd2 Lmnb1 Bard1 Ccnd1
E
E2F targets
ES: -0.41
NES:-1.88
F
E2F targets
Hmmr Cks1b Pole Hmgb3 Kpna2 Ccnb2 Cdkn3 Cks2 Gm10184 Depdc1a Cdc20 Cdk1 Lmnb1 Bard1 Birc5 Spag5 Kif2c Tcf19 Kif22 Kif4 Tk1 Aurka Cbx5 Ncapd2 Plk1 Mki67 Cdca3 Cenpe Top2a Racgap1 Chek2 Shmt1 Tacc3 H2ac18 H2aw Tubb1 Fhad1
G
Mitosis
ES: -0.39
NES:-1.86
H
Mitosis
Dnah7a Gsn Tuba1a Arhgef3 Cyth3 Klc1 Fgd1 Arfgef1 Taok1 Dock1 Wasl Ctnn Kif11 Cdk1 Anln Rabgap1l Dock4 Lats1 Cd2ap Plk1 Cenpe Tpx2 Hook1 Ccnb2 Kntc1 Rapgef6 Racgap1 Sorbs2 Fgd6 Top2a Nusap1 Bub1 Birc5 Prc1 Rasa2 Rictor Ect2 Cenpf Kif23 Lmnb1 Spag5 Kif2c Aurka Pif1 Kif22 Kif20b Kif4
I
Allograft rejection
ES: 0.42
NES:1.36
J
Allograft rejection
Gzme Ereg Ccnd1 Dyrk2 Wasl Inhbb Lif Il18rap Hif1a Pf4 Cxcl15 Ifngr1 Zap70 Il2rb Gzmb Gzma Ccl5 Cd8b1 Gbp2 Il2rg Cd40 Ccl19 Fas Cd3d Lck Cd2 Ccl11 Cd1d1 Ccl4 Srgn Csf1 H2-T23 Hck Psmb10 H2-Q7 Tnf Krt1
K
Interferon gamma response
ES: 0.40
NES:1.30
L
Interferon gamma response
Auts2 Lats1 Herc3 Rapgef6 Ifi27l2b Cd38 Mettl7b Casp3 Hif1a Ciita Ccl11 Bst1 Slamf7 H2-T23 Gzma Ccl5 Fibin Nfkbia Eif4e3 Ifitm1 Usp18 Bst2 Il2rb Fas Fgl2 Cd40 Ifi35 Cxcl10 Arl4a Ube2l6 C1ra Psmb10 H2-Q7 Psmb9 Tnfaip3 Gbp3
M
Fasl Irf7 H2-Q1 H2-K1 Ncr1 Prf1 Gzma Ccl5 Cxcl11 Irf8 H2-M5 Cxcl10 Ifng H2-Q10 Icosl H2-Ob Nkg7 Cxcl9 Gzmb Tnf H2-Q7 H2-T23 H2-M3 H2-T22 B2m H2-D1 H2-M2 Gzmc Irf4 Il2
dTAGV-1-3
dTAGV-1-2
dTAGV-1-1
Vehicle-2
Vehicle-1
N
Relative expression
DMSO
50 nM dTAGV-1
****
**
P = 0.052
CCL5
CXCL10
TNF

Supplemental Figure 3. Transcriptomic analysis reveals KRAS^{G12V} degradation triggers immune response signaling.

(**A**) Schematic showing the experimental design for transcriptomic analysis from *n* = 2 vehicle treated mice and *n* = 3 dTAG^{V}-1 treated mice. (**B**) Gene set enrichment analysis (GSEA) of differentially expressed genes showing the top positive and negatively enriched Hallmark pathways upon dTAG^{V}-1 treatment. (**C-H**) GSEA and heatmaps of the differentially expressed genes downregulated upon dTAG^{V}-1 treatment. Shown are G2M cell cycle (**C** and **D**), E2F targets (**E** and **F**) and mitosis (**G** and **H**), with the associated genes. (**I-L**) GSEA and heatmaps of the differentially expressed genes upregulated upon dTAG^{V}-1 treatment. Shown are allograft rejection (**I** and **J**) and interferon gamma response (**K** and **L**), with the associated genes. (**M**) Heatmap of the differentially expressed genes related to antitumor immunity in vehicle versus dTAG^{V}-1 treatment. (**N**) DMSO-normalized relative gene expression of AALE cells expressing FKBP12^{F36V}-KRAS^{G12V} treated with DMSO or 50 nM dTAG^{V}-1 for 48 h. Data is presented as mean ± s.d. of *n* = 3 technical replicates and are representative of *n* = 3 independent experiments. $^{**}P < 0.01$ and $^{****}P < 0.0001$ by a two-tailed Student's *t*-test.

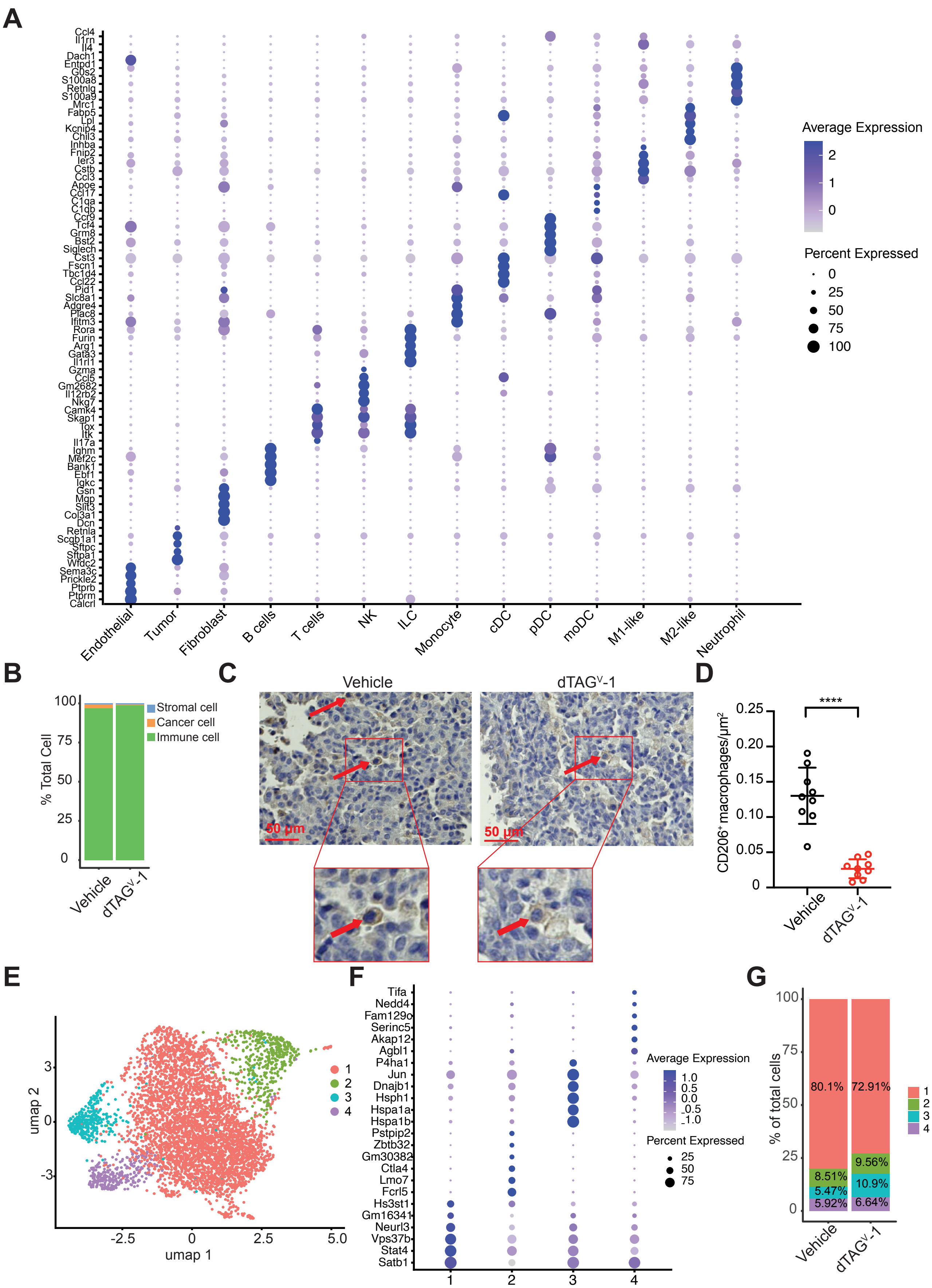

A
Ccl4
Il1rn
Il4
Dach1
Entpd1
G0s2
S100a8
Retnlg
S100a9
Mrc1
Fabp5
Lpl
Kcnip4
Chil3
Inhba
Fnip2
Ier3
Cstb
Ccl3
Apoe
Ccl17
C1qa
C1qb
Ccr9
Tcf4
Grm8
Bst2
Siglech
Cst3
Fscn1
Tbc1d4
Ccl22
Pid1
Slc8a1
Adgre4
Plac8
Ifitm3
Rora
Furin
Arg1
Gata3
Il1rl1
Gzma
Ccl5
Gm2682
Il12rb2
Nkg7
Camk4
Skap1
Tox
Itk
Il17a
Ighm
Mef2c
Bank1
Ebf1
Igkc
Gsn
Mgp
Slit3
Col3a1
Dcn
Retnla
Scgb1a1
Sftpc
Sftpa1
Wfdc2
Sema3c
Prickle2
Ptprb
Ptprm
Calcrl
Endothelial
Tumor
Fibroblast
B cells
T cells
NK
ILC
Monocyte
cDC
pDC
moDC
M1-like
M2-like
Neutrophil
Average Expression
2
1
0
Percent Expressed
0
25
50
75
100
B
% Total Cell
100
75
50
25
0
Stromal cell
Cancer cell
Immune cell
Vehicle
dTAGV-1
C
Vehicle
dTAGV-1
50 μm
50 μm
D
CD206+ macrophages/μm2
0.25
0.20
0.15
0.10
0.05
0
****
Vehicle
dTAGV-1
E
umap 2
3
0
-3
-2.5
0
2.5
5.0
umap 1
1
2
3
4
F
Tifa
Nedd4
Fam129c
Serinc5
Akap12
Agbl1
P4ha1
Jun
Dnajb1
Hsph1
Hspa1a
Hspa1b
Pstpip2
Zbtb32
Gm30382
Ctla4
Lmo7
Fcrl5
Hs3st1
Gm16341
Neurl3
Vps37b
Stat4
Satb1
1
2
3
4
Average Expression
1.0
0.5
0.0
-0.5
-1.0
Percent Expressed
25
50
75
G
% of total cells
100
75
50
25
0
80.1%
72.91%
8.51%
9.56%
5.47%
10.9%
5.92%
6.64%
Vehicle
dTAGV-1
1
2
3
4

Supplemental Figure 4. KRAS^{G12V} degradation reprograms the TME to promote anti-tumor immunity.

(**A**) Dot plot showing the top cell type specific marker genes identified in single-cell RNA-seq analyses. The size and opacity of the dots represent the percentage of cells and the average expression levels of genes in a cluster. (**B**) Distribution fraction of cancer, immune, and stromal compartments in response to indicated treatment. (**C**) Representative images of IHC staining for CD206 (MRC1) macrophages in the lung tumors after the indicated treatment. The scale bar represents 50 μm. (**D**) Quantification of CD206 staining after indicated treatment. Data is presented as mean ± s.d. of nine representative areas from n = 3 mice per group. ****$P < 0.0001$ by a two-tailed Student's t-test. (**E**) UMAP plot showing identified cell subsets in B cell population. (**F**) Dot plot showing the top cell type specific marker genes identified each subcluster of B cells. (**G**) Percentage of cells in the annotated B cell subsets in response to the indicated treatments.

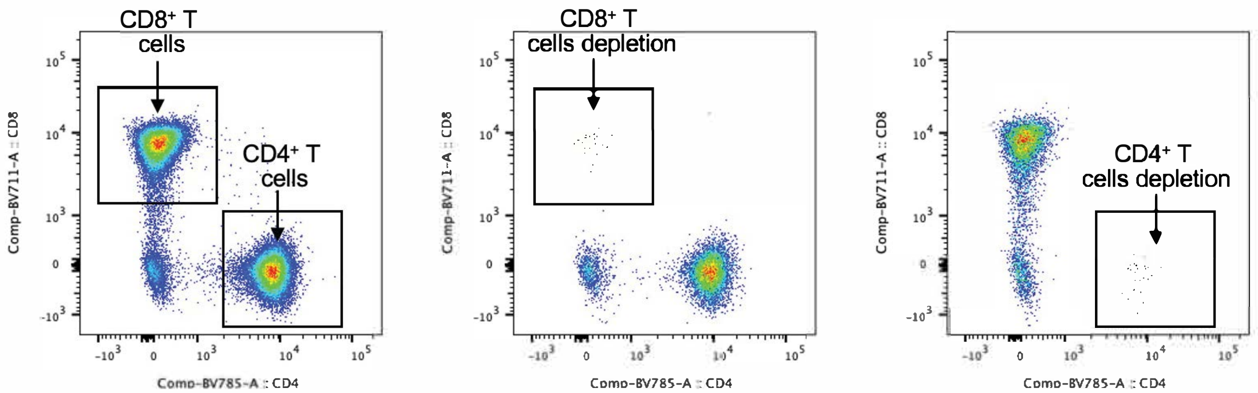

Supplemental Figure 5. Confirmation of CD8$^+$ and CD4$^+$ T cell depletion in the *in vivo* treatment study.

Representative density plot of flow cytometry analysis showing CD8$^+$ and CD4$^+$ T cell population in control and their depletion upon anti-CD8 or anti-CD4 treatment, respectively.

Methods

Cell culture

The following cell lines were employed in this study: NIH/3T3 (source: ATCC, media: DMEM with 10% calf serum and 1% Penicillin-Streptomycin), 293FT (source: Thermo Fisher Scientific, media: DMEM with 10% FBS and 1% Penicillin-Streptomycin), PATU-8902 (source: DSMZ, media: DMEM with 10% FBS and 1% Penicillin-Streptomycin), and AALE (source: Dr. William Hahn, Dana-Farber Cancer Institute; media: Lonza Small Airway Epithelial Cell Growth Medium BulletKit Cat#CC-3118). The development of engineered cell lines is detailed below. All cell lines were maintained in 37 ºC and 5% CO_2 incubators and routinely tested negative for mycoplasma contamination.

Lentiviral plasmid construction

Cloning of pLEX_305-dTAG-GFP and pLEX_305-dTAG-KRAS^{G12V} were previously described (1, 2). To generate pLEX_305-GFP and pLEX_305-KRAS^{G12V} gateway recombination cloning strategies (Invitrogen) were employed. pENTREGFP2 (Addgene Cat#22450) and pDONR225_KRAS4b_G12V were cloned into pLEX_305 (Addgene Cat#41390) using LR clonase (Thermo Fisher Scientific, Cat#11791-020) as previously described (1, 3).

Lentivirus production, transduction, and cell line generation

pLEX_305-GFP, pLEX_305-KRAS^{G12V}, pLEX_305-dTAG-GFP, and pLEX_305-dTAG-KRAS^{G12V} lentivirus was prepared and concentrated as previously described (1, 3).

NIH/3T3 GFP, KRAS^{G12V}, FKBP12^{F36V}-GFP, and FKBP12^{F36V}-KRAS^{G12V} cells

To generate NIH/3T3 GFP, KRAS^{G12V}, FKBP12^{F36V}-GFP, and FKBP12^{F36V}-KRAS^{G12V} cells, NIH/3T3 cells were transduced with pLEX_305-GFP, pLEX_305-KRAS^{G12V}, pLEX_305-dTAG-GFP, or pLEX_305-dTAG-KRAS^{G12V} concentrated lentiviral supernatant in the presence of 4 μg/mL polybrene and then selected with 2 μg/mL puromycin.

AALE FKBP12^{F36V}-GFP and FKBP12^{F36V}-KRAS^{G12V} cells

To generate AALE FKBP12^{F36V}-GFP and FKBP12^{F36V}-KRAS^{G12V} cells, AALE cells were transduced with pLEX_305-dTAG-GFP or pLEX_305-dTAG-KRAS^{G12V} concentrated lentiviral supernatant and then selected with 1.5 μg/mL puromycin.

PATU-8902 FKBP12^{F36V}-KRAS^{G12V}; KRAS$^{-/-}$ cells

Development and characterization of PATU-8902 FKBP12^{F36V}-KRAS^{G12V}; *KRAS*$^{-/-}$ cells were previously described (3, 4).

Confocal spinning disk microscopy

NIH/3T3 GFP or FKBP12^{F36V}-GFP cells were cultured on glass-bottom dishes (FluoroDish Cell Culture Dish, Cat#FD35) for 24 h and treated as indicated. After the indicated compound treatments, cells were fixed with 4 % paraformaldehyde in PBS for 15 minutes, washed three times with 1X PBS, and permeabilized with 0.1 % Triton X-100 in 1X PBS for 5 minutes. Cells were stained with nuclear counterstain DAPI (1:1000) for 5 minutes and washed three times with 1X PBS. Images were captured using a Dragonfly 200 High-speed Spinning Disk Confocal imaging platform (Andor Technology Ltd) with a Leica DMi8 microscope stand using a 100X/1.4 oil immersion objective, iXon EMCCD cameras and loaded with Fusion Version 2.3.0.36 (Oxford Instruments) software together with Imaris simultaneous deconvolution. The images were processed using ImageJ software.

Analysis of NIH/3T3 and AALE cell viability cultured as ultra-low adherent 3D-spheroids

NIH/3T3 and AALE cell viability was assayed in ultra-low adherent 3D-spheroid format using PrimeSurface 384-well 3D culture spheroid plates (S-bio, Cat#MS-9384WZ). NIH/3T3 cells were plated at a density of 250 cells/well in 50 µL media, and AALE cells were plated at a density of 900 cells/well in 50 µL media. After 24 hours of spheroid formation, cells were treated with compounds using the D300e digital dispenser (HP), and all wells were normalized to 100 nL total DMSO. 5 days later, plates were equilibrated to room temperature for 30 minutes followed by the addition of 10 µL CellTiter-Glo (Promega, Cat#G7570) for 15 minutes with shaking at room temperature. Luminescence was measured on the CLARIOstar Plus Plate Reader (BMG LabTech). The data was normalized to DMSO-treated control wells and analyzed using GraphPad PRISM v10.

Compounds

dTAG^V-1 (hydrochloride salt) was synthesized and formulated as previously described (3). In brief, dTAG^V-1 powder was brought to room temperature, and 5 mg was weighed out in each vial for daily injections. For 35 mg/kg administrations, dTAG^V-1 was formulated by first dissolving into DMSO (70 mg/mL) and vortexed for 30 seconds to completely dissolve the compound. Next, a 20 % solutol (Sigma, Cat#42966): 0.9 % sterile saline (Moltox, Cat#51-40S022.052) (w:v) solution was prepared and added to the 70 mg/mL dTAG^V-1 solution. To improve solubility, the formulation was heated at 45 °C for five-minute intervals with vortexing for 30 seconds. The final formulation contained 5 % DMSO / 20 % solutol / 75 % sterile saline. Formulations were prepared weekly and stored at room temperature. dTAG^V-1 was intraperitoneally administered.

Histology and immunohistochemistry

As previously described (5), tumor samples were collected fresh and directly fixed overnight in 4 % paraformaldehyde and then processed through graded ethanol, xylene, and into paraffin on a Leica Peloris automated tissue processor. Five-micron thick sections were prepared and either stained with hematoxylin (Leica, Cat#3801575) and eosin (Leica, Cat#3801619) on a Leica ST5020 stainer or immunostained on a Leica BondRX automated stainer, according to the manufacturer's instructions. In brief, tissues for immunostaining underwent deparaffinization online followed by epitope retrieval for 20 minutes at 100 °C with Leica Biosystems ER2 solution (pH 9, Cat#AR9640) and endogenous peroxidase activity blocking with hydrogen peroxide. Sections were incubated for 30 minutes at room temperature with the primary antibody as indicated: HA (Cell Signaling Technology, Cat#3724), pERK1/2 T202/Y204 (Cell Signaling Technology, Cat#9101), cleaved caspase-3 (Cell Signaling Technology, Cat#9579), Ki-67 (Abcam, Cat#ab16667), and CD206 (Abcam, Cat#ab64693). Primary antibodies were detected with antirabbit horseradish peroxidase-conjugated polymer and 3,3′-diaminobenzidine substrate that is provided in the Leica BOND Polymer Refine Detection System (Leica, Cat#DS9800). After counterstaining with hematoxylin, slides were scanned at 40X on a Leica AT2 whole slide scanner for analysis (Aperio Image Library v12.0.16, Leica Biosystems) or using a Keyence fluorescence microscope (BZ-X Series). The images were processed and quantified using ImageJ software.

Multiplex image analysis

The details of the procedure have been reported previously (6). Briefly, separate opal detector fluorophores were utilized for each different phenotyping marker as indicated: CD19 (Abcam, 1:1000, Cat#ab245235)/Opal520, CD3 (Abcam, 1:300, Cat#ab16669)/Opal780, and Foxp3 (Cell Signaling Technology, 1:200, Cat#98377S)/Opal690. Nuclear stain was carried out with spectral DAPI in the final step. Upon completion of scanning on the Vectra (Perkin Elmer), regions of interest (ROIs) were selected from whole slide scans using Phenochart and unmixed on inForm Tissue Analysis software (Akoya Biosciences). QuPath software was used for quantification analysis (7). QuPath enables cell detection using the nuclear stain, DAPI, and segments each individual cell. For phenotyping of individual cells, machine learning approach was utilized to

generate an object classifier for each marker, CD19, CD3 and Foxp3, which was then compiled together and applied onto multiplex images, subsequently normalized to the area size of tumor (per 100 μm^2).

Western blotting

Cells were lysed in RIPA buffer supplemented with cOmplete protease inhibitor (Roche), PhosSTOP phosphatase inhibitor (Roche), and 0.1% Benzonase (Millipore Sigma) at 4 °C with gentle end-to-end rotation for 1 hour. Similarly, tumor nodules were collected and homogenized in RIPA lysis buffer supplemented with Halt™ Protease and Phosphatase Inhibitor Cocktail for protein extraction. Lysates were clarified by centrifugation at 20,000 xg for 10 minutes at 4 °C. Subsequently, an equal amount of protein samples was subjected to 4-12% gradient gel SDS-PAGE, followed by overnight incubation with the primary antibody as indicated: HA (Cell Signaling Technology, Cat#3724), GFP (Cell Signaling Technology, Cat#2555), α-Tubulin (Cell Signaling Technology, Cat#2125), total ERK1/2 (Cell Signaling Technology, Cat#9102), pERK1/2 T202/Y204 (Cell Signaling Technology, Cat#9101), MEK1/2 (Cell Signaling Technology, Cat#4694), pMEK1/2 S217/221 (Cell Signaling Technology, Cat#9121), KRAS (LS Bio, Cat#LS-C175665), and Actin (Sigma, Cat#A5441). The next day, the membrane was washed with TBS-T (TBS with 0.1% Tween) and incubated with appropriate secondary antibodies (LI-COR) at room temperature for 1 hour. Membranes were imaged using the LI-COR Odyssey® Imaging System.

Quantitative PCR

Cells were treated, lysed, and homogenized using QIAshredder columns (Qiagen, Cat#79654), and RNA was extracted using the RNeasy Plus Mini Kit (Qiagen, Cat#174134), according to the manufacturer's instructions. RNA concentration was determined using CLARIOstar Plus Plate Reader with LVIS plate (BMG LabTech, Cat#1600-680-102). cDNA was synthesized using the iScript Advanced cDNA synthesis kit (BioRad, Cat#1725038), according to the manufacturer's instructions. TaqMan qPCR was performed using QuantStudio 5 (Applied Biosystems). The relative expression of all targets including *RPS17* (Thermo Fisher Scientific, Cat#4453320 Hs00734303_g1), *CCL5* (Thermo Fisher Scientific, Cat#4453320 Hs00982282_m1), *CXCL10* (Thermo Fisher Scientific, Cat#4453320 Hs00171042_m1), and *TNF* (Thermo Fisher Scientific, Cat#4453320 Hs00174128_m1) was calculated using the $2^{-\Delta\Delta Cq}$ method.

MRI quantification

Animals were anesthetized with isoflurane to perform MRI of the lung field using Biospec 3T Magnet system (Bruker) to scan 16 consecutive sections. Tumor volumes within the whole lung were quantified using 3-D slicer software to reconstruct MRI volumetric measurements as described previously (8).

Tumor-infiltrating immune cell profiling

Tumor-bearing mice were euthanized, and mouse lungs were cut and minced into small pieces followed by digestion in collagenase D and Dnase I in Hank's balanced salt solution at 37 °C for 30 minutes. As previously described (9), the digested tissue was then subjected to a 70 µm cell strainer and treated with 1×RBC lysis buffer. The cell pellets were subsequently stained with indicated markers and then resuspended in PBS with 2% FBS for flow cytometry analysis on BD LSRFortessa and analyzed using FlowJo software. The following flow antibodies were used as indicated: CD45 (BioLegend, Cat#103155), CD8 (BioLegend, Cat#100759), CD44 (BioLegend, Cat#103032), CD62L (BioLegend, Cat#104417), CD69 (BioLegend, Cat#104508), and GZMB (BD Biosciences, Cat#562462).

RNA extraction and bulk-RNA sequencing analysis

Tumor nodules were collected and homogenized, and then subjected to total RNA extraction using RNeasy Plus Mini Kit (Qiagen, Cat#74136) according to the manufacturer's instructions. Read qualities were evaluated using FASTQC (Babraham Institute) and mapping to GRCm38 (GENCODE M25) reference genome using STAR program (10). Gene expressions were quantified using RSEM program (11). Identification of differentially expressed genes was performed using DESeq2 in R/Bioconductor (R version 4.0.4). All plots were generated using customized R scripts.

Gene set enrichment analysis

Pathway enrichment analysis was performed on all genes ranked from high to low by DESeq2 estimated log2 fold change using the GSEAPreRanked function available in gene set enrichment analysis (GSEA) program with enrichment statistic classic and 1,000 permutations (12). Gene sets (hallmark) were downloaded from MsigDB (13). Differential expression genes involved in top enriched pathways were selected to generate heatmaps using pheatmap R function with default hierarchical clustering method for gene orders. For large pathways, genes were truncated based on absolute log2 fold change values for visualization purposes. Additionally, a customized gene set including genes associated with antigen presentation (*B2m*), interferon signaling (*Irf4*, *Irf7*, and *Irf8*), cytotoxic granzymes (*Gzma*, *Gzma* and *Gzmc*), NK cells (*Nkg7* and *Ncr1*), and antitumor cytokines/chemokines (*Tnf*, *Ifng* and *Cxcl10*) was established and a heatmap was generated based on their normalized expression values.

Single-cell RNA sequencing and analysis

Single-cell RNA-seq (scRNA-seq) was performed as previously described (8). To account for interindividual variability, we harvested and pooled fresh tumor-bearing lungs from two mice each group. Single-cell suspensions were obtained as above in the Tumor-infiltrating Immune Cell Profiling section and were sorted using 4′,6-diamidino-2-phenylindole (DAPI) staining. Cells were then resuspended into single cells and loaded onto a 10X Genomics Chromium instrument to generate single-cell gel beads in emulsion. Approximately 5,000-10,000 cells were loaded per channel. Single-cell RNA sequencing (scRNA-seq) libraries were prepared using the following Single Cell 3′ Reagent Kits following the Single Cell 3′ Reagent Kits V.2 User Guide. Libraries were run on an Illumina HiSeq 4000 system.

scRNA-seq data processing was described in our previous study (8). Briefly, Cell Ranger Single-Cell Software Suite was used to perform sample de-multiplexing, mapping (GRCm38 genome reference), barcode processing, and gene expression quantification. Filtered gene-barcode matrices that contained only barcodes with unique molecular identifier (UMI) counts that passed the threshold for cell detection were imported into R as a Seurat (Version 4.1.0) object for further analysis (14, 15). Cells with less than 200 genes detected or greater than 5% mitochondrial RNA content were deemed as low quality and excluded from analysis. RNA counts were normalized using Seurat::SCTransform function with regressions of cell cycle score, ribosomal and mitochondrial percentages for each sample. Multiple samples were integrated using Seurat standard scRNSeq integration workflow with 3,000 anchor genes. A shared nearest neighbor graph was then built based on the first 40 principal components followed by identification of cell clusters using Leiden algorithm. The same principal components were used to generate the UMAP projection (16). Cell types were manually annotated based on canonical cell type markers and differential expressed genes of each cluster identified using Seurat::FindAllMarkers function with a logistic regression model.

References

1. Nabet B, et al. The dTAG system for immediate and target-specific protein degradation. *Nat Chem Biol.* 2018;14(5):431-441.
2. Seong B K A, et al. TRIM8 modulates the EWS/FLI oncoprotein to promote survival in Ewing sarcoma. *Cancer Cell.* 2021;39(9):1262-1278 e1267.
3. Nabet B, et al. Rapid and direct control of target protein levels with VHL-recruiting dTAG molecules. *Nat Commun.* 2020;11(1):4687.
4. Ferguson F M, et al. Discovery of a selective inhibitor of doublecortin like kinase 1. *Nat Chem Biol.* 2020;16(6):635-643.
5. Zhang H, et al. Histone deacetylase 6 inhibition exploits selective metabolic vulnerabilities in LKB1 mutant, KRAS driven NSCLC. *J Thorac Oncol.* 2023;18(7):882-895.
6. Ruffin A T, et al. B cell signatures and tertiary lymphoid structures contribute to outcome in head and neck squamous cell carcinoma. *Nat Commun.* 2021;12(1):3349.
7. Bankhead P, et al. QuPath: open source software for digital pathology image analysis. *Sci Rep.* 2017;7(1):16878.
8. Zhang H, et al. CDK7 inhibition potentiates genome instability triggering anti-tumor immunity in small cell lung cancer. *Cancer Cell.* 2020;37(1):37-54 e39.
9. Pan Y, et al. Nemvaleukin alfa, a novel engineered IL-2 fusion protein, drives antitumor immunity and inhibits tumor growth in small cell lung cancer. *J Immunother Cancer.* 2022;10(9).
10. Dobin A, et al. STAR: ultrafast universal RNA-seq aligner. *Bioinformatics.* 2013;29(1):15-21.
11. Li B, and Dewey C N. RSEM: accurate transcript quantification from RNA-Seq data with or without a reference genome. *BMC Bioinformatics.* 2011;12:323.
12. Subramanian A, et al. Gene set enrichment analysis: a knowledge-based approach for interpreting genome-wide expression profiles. *Proc Natl Acad Sci U S A.* 2005;102(43):15545-15550.
13. Liberzon A, et al. Molecular signatures database (MSigDB) 3.0. *Bioinformatics.* 2011;27(12):1739-1740.
14. Stuart T, et al. Comprehensive integration of single-cell data. *Cell.* 2019;177(7):1888-1902 e1821.
15. Hao Y, et al. Integrated analysis of multimodal single-cell data. *Cell.* 2021;184(13):3573-3587 e3529.
16. McInnes L, et al. UMAP: uniform manifold approximation and projection for dimension reduction. *arXiv:180203426.* 2020.
