# Supplementary figures and images for: Targeted degradation of oncogenic KRASG12V triggers antitumor immunity in lung cancer models
Source: J Clin Invest. 2024 Dec 24;135(2):e174249. doi: 10.1172/JCI174249 (PMC11735103; doi:10.1172/JCI174249)

Figure 1C unedited blots

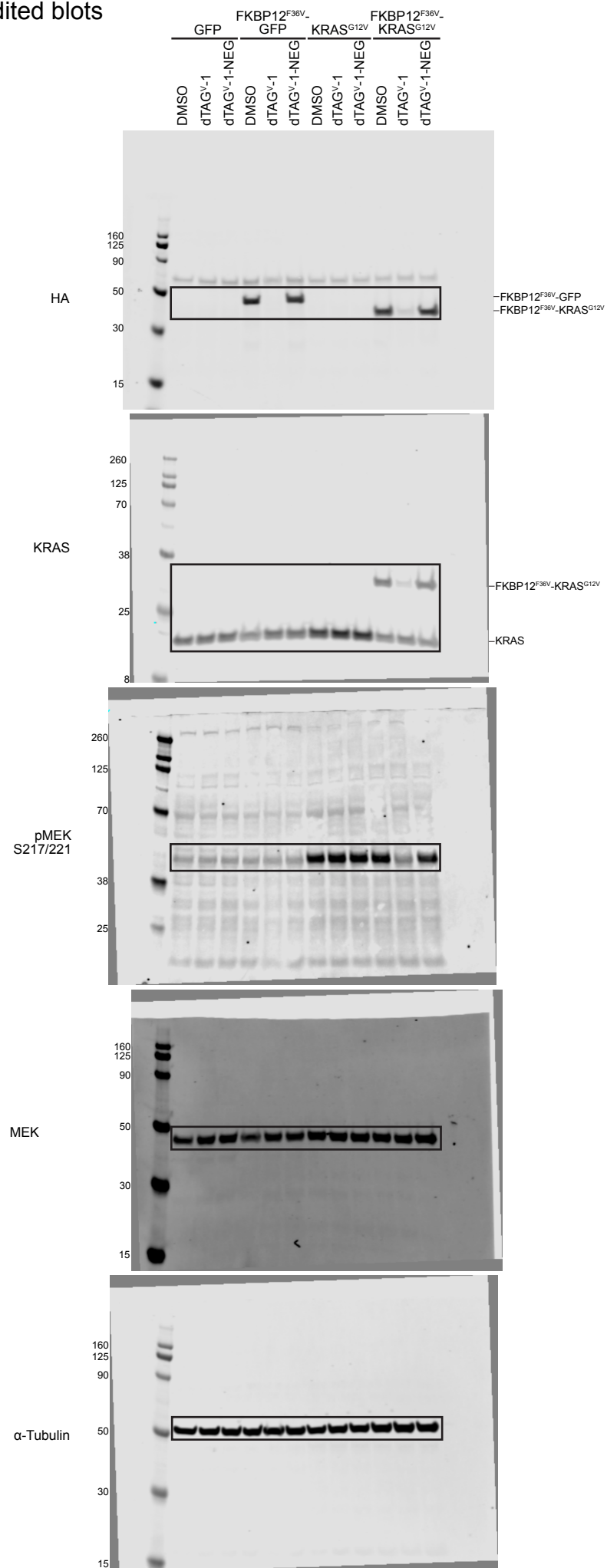

Figure 3B unedited blots

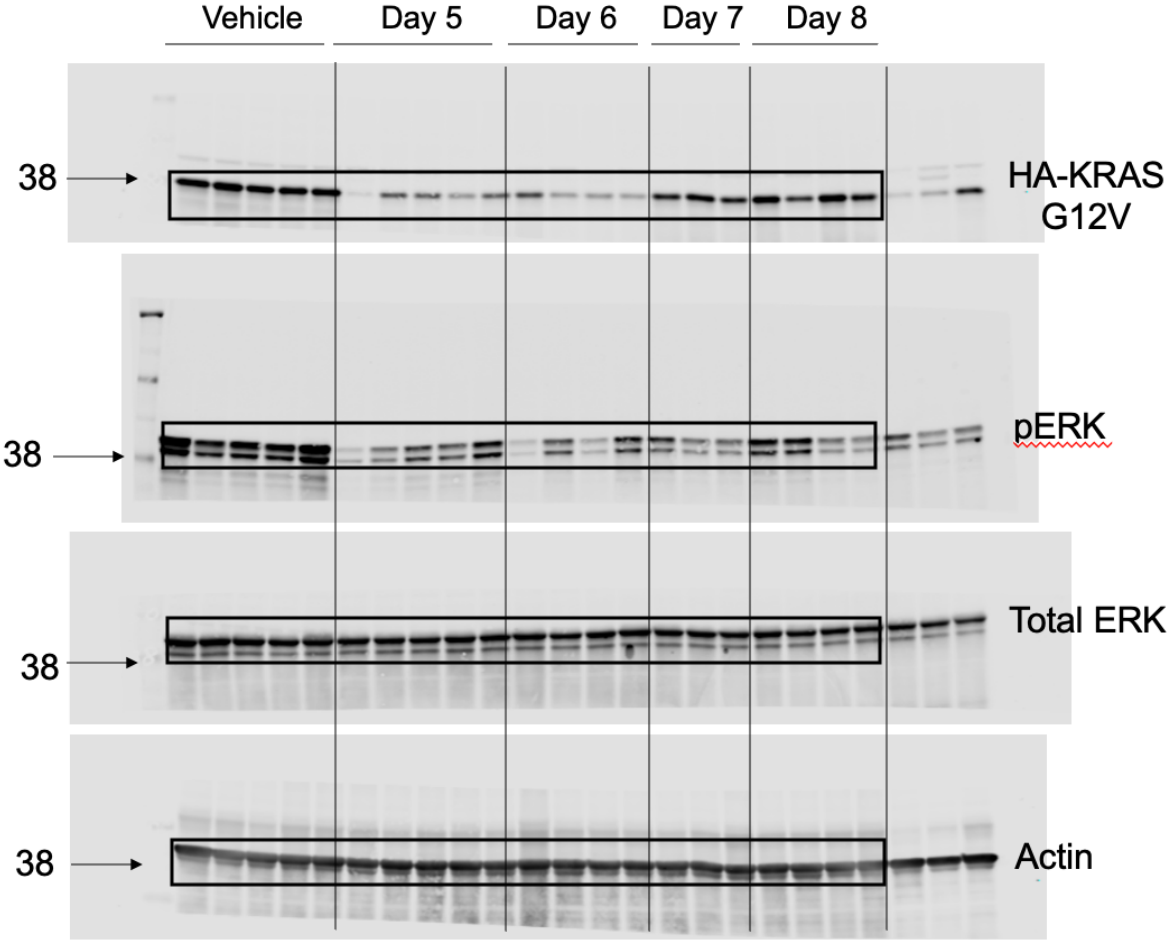

Supplemental Figure 1B unedited blots

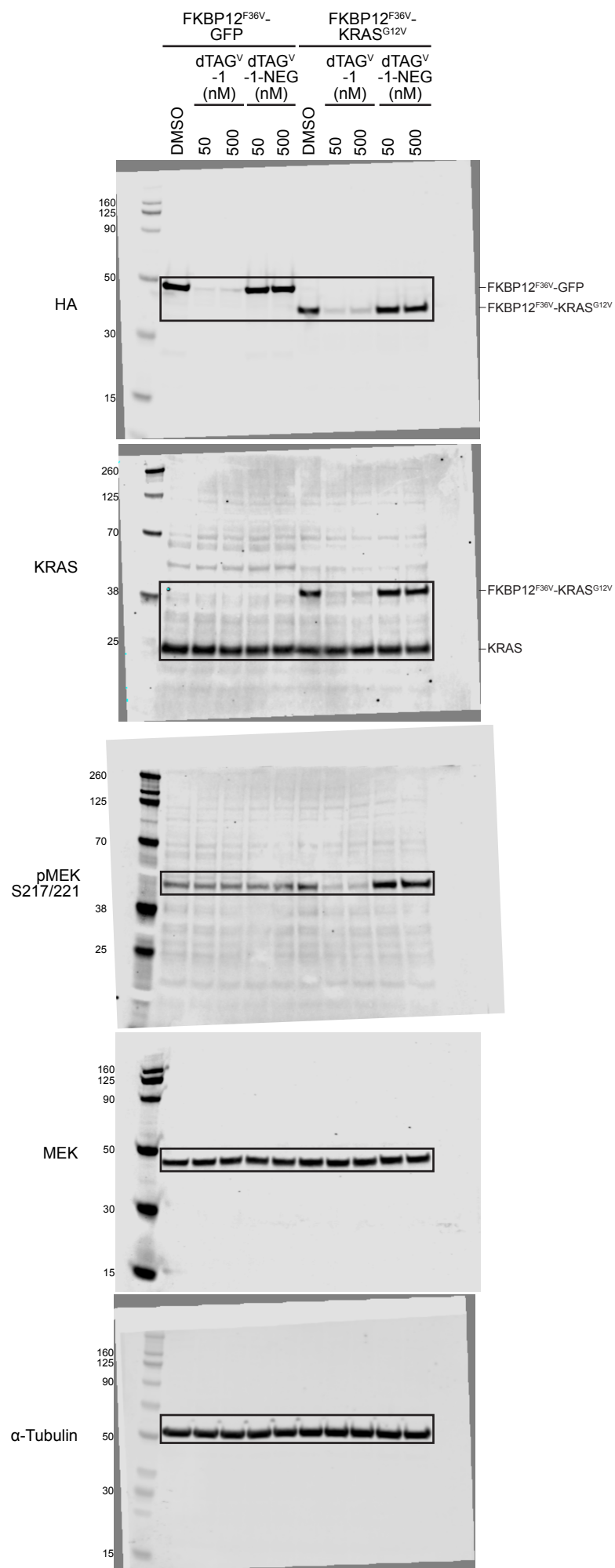

Supplement: Unedited blot and gel images [file jci-135-174249-s064.pdf]
